# Deimmunization for gene therapy: host matching of synthetic zinc finger constructs enables long-term mutant Huntingtin repression in mice

**DOI:** 10.1186/s13024-016-0128-x

**Published:** 2016-09-06

**Authors:** Carmen Agustín-Pavón, Michal Mielcarek, Mireia Garriga-Canut, Mark Isalan

**Affiliations:** 1 Department of Life Sciences, Imperial College London, London, SW7 2AZ UK; 2 Cell and Developmental Biology Program, Centre for Genomic Regulation (CRG), Dr. Aiguader 88, 08003 Barcelona, Spain; 3 Universitat Pompeu Fabra (UPF), Barcelona, Spain; 4 Current address: Predepartmental Unit of Medicine, Faculty of Health Sciences, University Jaume I, Av. de Vicent Sos Baynat, s/n 12071 Castelló de la Plana, Spain

**Keywords:** Monogenetic disease, Gene therapy, Huntington’s disease, Neurodegenerative disorder, Immune response, Synthetic transcription factors, rAAV, Host optimization

## Abstract

**Background:**

Synthetic zinc finger (ZF) proteins can be targeted to desired DNA sequences and are useful tools for gene therapy. We recently developed a ZF transcription repressor (ZF-KOX1) able to bind to expanded DNA CAG-repeats in the huntingtin (*HTT*) gene, which are found in Huntington’s disease (HD). This ZF acutely repressed mutant *HTT* expression in a mouse model of HD and delayed neurological symptoms (clasping) for up to 3 weeks. In the present work, we sought to develop a long-term single-injection gene therapy approach in the brain.

**Method:**

Since non-self proteins can elicit immune and inflammatory responses, we designed a host-matched analogue of ZF-KOX1 (called mZF-KRAB), to treat mice more safely in combination with rAAV vector delivery. We also tested a neuron-specific enolase promoter (pNSE), which has been reported as enabling long-term transgene expression, to see whether *HTT* repression could be observed for up to 6 months after AAV injection in the brain.

**Results:**

After rAAV vector delivery, we found that non-self proteins induce significant inflammatory responses in the brain, in agreement with previous studies. Specifically, microglial cells were activated at 4 and 6 weeks after treatment with non-host-matched ZF-KOX1 or GFP, respectively, and this was accompanied by a moderate neuronal loss. In contrast, the host-matched mZF-KRAB did not provoke these effects. Nonetheless, we found that using a pCAG promoter (CMV early enhancer element and the chicken β-actin promoter) led to a strong reduction in ZF expression by 6 weeks after injection. We therefore tested a new non-viral promoter to see whether the host-adapted ZF expression could be sustained for a longer time. Vectorising mZF-KRAB with a promoter-enhancer from neuron-specific enolase (*Eno2*, rat) resulted in up to 77 % repression of mutant *HTT* in whole brain, 3 weeks after bilateral intraventricular injection of 10^10^ virions. Importantly, repressions of 48 % and 23 % were still detected after 12 and 24 weeks, respectively, indicating that longer term effects are possible.

**Conclusion:**

Host-adapted ZF-AAV constructs displayed a reduced toxicity and a non-viral pNSE promoter improved long-term ZF protein expression and target gene repression. The optimized constructs presented here have potential for treating HD.

**Electronic supplementary material:**

The online version of this article (doi:10.1186/s13024-016-0128-x) contains supplementary material, which is available to authorized users.

## Background

The development of increasingly safe gene therapy vectors, with reduced immunogenicity [1], low insertional capabilities [2], and new and more effective delivery strategies [3, 4], has led to several successful clinical trials. Examples include a therapy against metachromatic leukodystrophy (inserting the functional enzyme arylsulfatase A in hematopoietic cells) [5], and a breakthrough in AIDS treatment that promises a 'functional cure' for HIV [6]. In particular, the latter study employed the technology of synthetic zinc finger nucleases [7], targeted to knock-out the CCR5 receptor in CD4 T cells, *ex vivo*. Nuclease-modified cells were autologously transplanted back into patients, achieving drug-free reduction of viraemia. Site-specific nuclease technology is highly scaleable and, with the advent of vectorisable RNA-programmable nucleases such as CRISPR/Cas9 lentiviruses [8], a revolution in genome editing is underway.

Despite this progress, it is becoming clear that the host immune system is a major barrier to successful long term therapies. In some cases, including the examples above, cells can be treated *ex vivo*, or with a single short intervention. However, in many diseases it is necessary to modify the expression of disease genes in vivo. The superbly versatile CRISPR/Cas systems face an issue here, because of their bacterial origins: their immunogenicity limits their use in repetitive or sustained dosage regimes. Even zinc finger nucleases use bacterial nuclease domains (*FokI* [9]) and it is unclear how they would be tolerated in vivo.

Immunological effects are particularly relevant when considering gene therapies for neurodegenerative diseases. Most neurodegenerative diseases require the correction of mutation(s) in vivo, directly in the affected tissue, or the sustained expression of therapeutic factors [10]. Since the brain has limited regenerative capacity, and complex connectivity, the tissue cannot simply be removed, repaired and re-implanted. Furthermore, a collection of recent articles has demonstrated delayed immune responses when injecting foreign proteins from AAV vectors into the brain parenchyma [11-13]. Strikingly, despite the immune-privileged environment of the brain, even GFP can induce a strong inflammatory and immune response in both rats and monkeys [11, 12]. Similarly, a human enzyme with potential use in Parkinson’s disease therapy has unwanted effects in rats [11]. Using the vector AAV9, which is capable of infecting both neurons and glial cells, neuronal death starts as early as 3 weeks after injection. Therefore, even though a range of new generation synthetic biology tools are being developed for degenerative diseases [10], the brain still remains a challenging target for gene therapy.

We recently developed a zinc finger-based gene therapy approach to target the fatal monogenic neurodegenerative disorder Huntington's disease (HD). To be successful, this project requires tackling the problems of sustained transgene expression in the brain [14]. In HD, the huntingtin (*HTT*) gene is expanded in a region containing repeats of the glutamine-encoding DNA triplet, CAG. This results in expanded poly(CAG) transcripts and polyglutamine (polyQ) proteins, which are both cytotoxic [15]. Neurodegeneration occurs in several motor-related areas of the brain, leading to symptoms that include psychiatric disturbances, chorea, and eventually fatality. Nine human genes contain potential CAG-expansion regions and every one is associated with a polyQ disease [16]. The available pharmacological interventions only alleviate symptoms, without stopping disease progression, and so new treatments are desperately required.

Several new therapeutic approaches in animal models of HD are beginning to show impressive results, especially using antisense oligonucleotides [17, 18]. These approaches allow the regulation of the gene product, but cannot correct the mutation or stop transcription. Unfortunately, in vivo mutation correction - using genome-editing tools directly in the brain - is not easily achievable. We found that zinc finger nucleases do not cleave poly(CAG) DNA efficiently even in vitro, presumably because the highly repetitive DNA prevents correct nuclease dimerization. By contrast, we showed that long-chain zinc fingers, fused to a KRAB transcription repression domain, were a feasible alternative [14]. Since its discovery [19], the human KRAB domain from KOX1 (ZNF10) has proved to be an efficient repressor of target genes, when fused to heterologous DNA-binding domains [20]. KOX1 recruits cellular factors that lay down heterochromatin and thus cause strong long-term and long-range gene repression across a genomic locus [21].

Taking this KRAB-repressor approach, and aiming for a treatment that could potentially be used to treat all the nine polyQ diseases, we designed a synthetic ZF-KOX1 fusion to bind expanded CAG repeats selectively [14]. We showed that intracerebral administration of ZF-KOX1, in recombinant adeno-associated vectors (pseudotype rAAV2/1), was able to acutely reduce the load of mutant huntingtin (*mut HTT*) RNA, and HTT protein, in the striata of R6/2 mice. The reduction was greatest at 2 weeks after treatment and was paralleled by a delay in the onset of clasping behaviour (a sign of neurological degeneration). These results demonstrated that synthetic transcription factors are therapeutically active after viral delivery in the brain.

In the current study, we aimed to test the consequences of expressing a synthetic zinc finger peptide in the brain over a longer period, to check for inflammatory and innate immune responses against non-host proteins. In particular, although the zinc finger scaffold we used is based on the mouse Zif268 protein [22], the DNA-recognition helices were not originally designed to minimise the number of foreign (non-mouse) peptide sequences, and these are potentially antigenic. Moreover, the human KOX1 (ZNF10) gene does not have an exact mouse homolog. Consequently, to avoid possible immune rejection in the mouse, we designed a new mouse host-matched ZF (mZF) that aimed to minimise the number of foreign peptide sequences that might form epitopes. For example, we replaced KOX1 with a KRAB domain from the mouse *Zfp87* gene (also called *Mzf22* [23]) to make mouse host-adapted mZF-KRAB. We injected ZF-KOX1, mZF-KRAB or GFP into the brains of mice and evaluated inflammatory responses and neuronal loss, initially up to 6 weeks after treatment, while comparing *mut HTT* repression efficiency. We chose this time point because it precedes full symptom development in our mouse model, thus minimizing animal suffering, while still allowing time for inflammatory responses to develop: previously reported results showed that inflammatory responses were well developed five weeks post-injection of AAV in rats [11]. Having evaluated these responses, we went on to test our new lead construct, mZF-KRAB, with a different, non-viral promoter from neuron-specific enolase (*Eno2*, rat). The aim was to see whether we could achieve repression of the mutant HTT allele over a longer time period. Specifically, we aimed to verify whether there was measurable repression in the whole brain for at least 6 months after AAV injection. Thus, we aimed to develop the basis for a single-injection, long-term gene therapy for HD.

## Methods

### Design of the host-adapted ZF

**Fig. 1:**
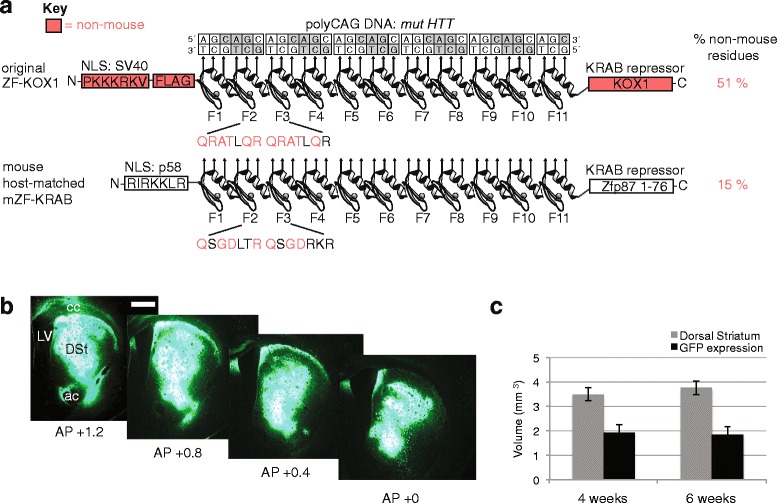
Zinc finger (ZF) mouse host-adaptation design and rAAV2/1 transfer into the striatum. **a** Comparison of the ZF-KOX1 and mZF-KRAB zinc finger repressor designs, showing the 11-finger constructs aligned to their target poly(CAG) DNA sequence (*mut HTT*). Protein domains containing non-mouse peptide sequences (containing potential foreign epitopes) are shaded in red. The sequences of representative DNA recognition helices from fingers 2 and 3 (F2, F3) are displayed below the ZF arrays, with foreign sequences in red font. The percentage totals of non-mouse residues within the full length peptide sequences are given to show that the mouse-adapted design reduces overall non-host sequences. Full annotated sequences are provided in Additional file 1. **b** Representative specimen showing GFP expression in mouse coronal slices of hemi-brains, from anterior to posterior view. The anteroposterior (AP) location of each slice within the brain of the mouse is shown as AP distance from Bregma, following [39]. **c** Bar chart showing the average volume (±S.E.M) of the whole dorsal striatum and the volume covered by GFP fluorescence. Abbreviations: NLS, nuclear localization signal; ac, anterior commissure; cc, corpus callosum; DSt, dorsal striatum; LV, lateral ventricle. Scale bar: 1 mm

Full sequences are available in Additional file 1. We converted ZF-KOX1 into a more mouse-compatible version, mZF-KRAB, as follows: (1) We removed the triple FLAG-tag reporter from ZF-KOX1. (2) We replaced the viral SV40 nuclear localization signal (NLS) with a mouse primase p58 NLS (RIRKKLR; GenBank: BAA04203.1), using native adjacent residues as linkers. (3) We employed a zinc finger framework that was as close as possible to the mouse Zif268 sequence [22], while retaining functional CAG-binding residues on the DNA recognition helices. Thus, the QRATLQR helix was changed to QSGDLTR or QSGDRKR (differences to wild-type mouse protein sequence vary from finger-to-finger within the Zif268 scaffold; Fig. 1a). Previous phage ELISA experiments [24], guided our helix design to bind CAG triplets. (4) We modified the ZF linkers to make them as close as possible to canonical ZF linkers (e.g., TGEKP, TGQKP), while retaining non-wild-type longer spacers (TG**S**QKP) after every 2 fingers; these spacers are essential for long ZF arrays to function [25]. Longer linkers after Fingers F5 and F11 were also slightly shortened (see Additional file 1). (5) We replaced human KOX1 with the mouse KRAB repression domain from Zfp87 (a.k.a. Mzf22 [23]; refSeq_NM_133228.3). This domain was chosen because the 1-76 amino acid KRAB-domain fragment of Zfp87, when fused to Gal4 DNA-binding domain, has been reported to have similar levels of repression compared to Gal4-KOX1 [23].

### Mice

For this study we used R6/1, R6/2 and wild type (WT) mice. R6/2 transgenic mice were purchased form Jackson Laboratories (B6CBA-Tg(HDexon1)62Gpb/3 J). Ovarian transplanted hemizygous females and WT B6CBAF1/J males were bred in house, and progeny was genotyped as previously described [26]. R6/1 transgenic mice (B6.Cg-Tg(HDexon1)61Gpb/J) and WT controls (C57BL/6 J) were purchased form Jackson Laboratories. For the testing of mZF-KRAB, we decided to switch from R6/2 to R6/1 to avoid early onset of symptoms and to comply with animal welfare conditions in the UK, since the aim of this study was not to check for a phenotype reversal. Stereotaxic injections were performed on 4-week-old R6/2 mice, 8-week-old R6/1 mice and 4- to 8-week-old WT mice. All animal experiments were conducted in accordance with Directive 86/609/EU of the European Commission, the Animals (Scientific Procedures) 1986 Act of the United Kingdom, and following protocols approved by the Ethical Committee of the Barcelona Biomedical Research Park and the Animal Welfare and Ethical Review Body of Imperial College London. The number of mice for each experiment is given in Additional file 2.

### Husbandry, genotyping and CAG repeat sizing

Hemizygous R6/1 mice were purchased from Jax Laboratories and bred by backcrossing R6/1 males to (CBA x C57BL/6) F1 females (B6CBAF1/OlaHsd, Charles River, UK). Genomic DNA was isolated from a tail tip. R6/1 mice were genotyped by PCR and the CAG repeat was measured by sequencing as previously described [27].

### Production of rAAV

rAAV2/1-GFP, rAAV2/1–ZF–KOX1 and rAAV2/1–mZF–KRAB, containing a pCAG promoter (CMV early enhancer element and the chicken β-actin promoter) and Woodchuck hepatitis virus postranscriptional regulatory element (WPRE) [28], were produced at the Centre for Animal Biotechnology and Gene Therapy of the Universitat Autonoma of Barcelona, as described previously [14]. Recombinant virus was purified by precipitation with PEG 8000, followed by iodixanol gradient ultracentrifugation with final titers of ~10^12^ genome copies/mL. rAAV2/1–pNSE-mZF–KRAB-WPRE and rAAV2/9–pHSP-mZF–KRAB-WPRE were produced in a similar manner.

### Stereotaxic surgery

Briefly, mice were anesthetized with a mix of ketamine (75 mg/kg) and medetomidine (1 mg/kg, i.p.) or isoflurane (preferred) and fixed on a stereotaxic frame. Analgesia was provided by buprenorphine (8 μg/kg, s.c.). AAVs were injected unilaterally into the striatum (A/P +0.7 mm, M/L ± 1.8 mm, D/V −2.5 mm, relative to bregma) using a 10 μl Hamilton syringe at a rate of 0.25 μl/min, controlled by an Ultramicropump (World Precision Instruments). In all experiments, except where noted, for each hemisphere we administered 2 × 10^9^ genomic particles or sterile PBS. We assume that the efficiency of transduction is similar in all groups because infections spread throughout the striata (Fig. 1b, c). However, we note that different volumes were used to match viral titre between all samples (3 μl injections for ZF-KOX; 1.5 μl injections for PBS, GFP and mZF-KRAB). Mice were killed at 2, 4 or 6 weeks after the injections, for qRT-PCR and histological analyses. To minimise animal use, the data at 2 weeks were taken from a subset of mice from our previous study [14].

### Intraventricular AAV injection protocol

Neonatal mice (P0.5) were cryoanesthetized (3 weeks dataset) or anesthetized with isoflurane (6, 12 and 24 weeks datasets) and subjected to bilateral intraventricular injection of AAVs, within 24 h of birth to ensure full ventricular dilation, using a protocol slightly modified from [29]. The maximum possible volume of 2 μl of viral vector, or PBS, was injected into each cerebral lateral ventricle, using a sterile Hamilton microsyringe. Thus, we injected 4 μl in total per mouse, with a viral titre of 2.5 x 10^9^ AAV / μl (10^10^ viral particles total). Mice were killed at 3, 6, 12 or 24 weeks after the injections and brains were harvested, snap frozen in liquid nitrogen, and stored at −80 C until further analysis by qRT-PCR.

### qRT-PCR for 2, 4 and 6 weeks dataset

Mice were humanely killed by cervical dislocation. As rapidly as possible, they were decapitated and the striata were dissected on ice and immediately frozen in liquid nitrogen, for later RNA extraction. RNA was prepared with RNeasy kit (Qiagen) and reverse transcribed with Superscript II (Invitrogen). Real Time PCR was performed in a LightCycler® 480 Instrument (Roche) using LightCycler® 480 SYBR Green I Master (Roche). SYBR Advantage GC qPCR Premix (Clontech) was used to amplify the human *HTT* transgene in R6/2 and R6/1 templates. For technical replicates, each PCR was done at least in triplicate, and results normalized to three housekeeping genes (*mHprt*, *mActb* and *mAtp5m* as in our previous study [14]). At least 3 independent biological replicates were done for each experiment. Primer sets are given in full in Additional file 3.

### qRT-PCR for 3, 6, 12 and 24 weeks dataset

Total RNA was extracted with the mini-RNA kit according to the manufacturer’s instructions (Qiagen). Reverse transcription (RT) was performed using Superscipt III reverse transcriptase (Invitrogen) and a mixture of Oligo-dT and random hexamers (Invitrogen). The final RT reaction was diluted 10-fold in nuclease-free water (Sigma) for further qPCR reactions. All Taqman-qPCR reactions were performed using the LightCycler 480 Real-Time PCR Detector (Roche) as described [27]. Stable housekeeping genes for qPCR profiling were determined using the Primer Design geNorm™ Housekeeping Gene Selection Mouse Kit with PerfectProbe™ software (Additional file 4). The following housekeeping genes were identified as suitable for qPCR analysis: *B2m*, (Beta-2-microglobulin, 12010), *18S* (18S rRNA, 19791), *Eif4A2* (Eukaryotic translation initiation factor 4A2, 13682). Estimation of mRNA copy number was determined in triplicate for each RNA sample by comparison to the geometric mean of three endogenous housekeeping genes (Primer Design) as described previously [27]. Primer sets are given in full in Additional file 3.

### Immunohistochemistry

Mice were transcardially perfused with PBS followed by formalin 4 % (v/v). Brains were removed and post-fixed overnight at 4 °C in formalin 4 % (v/v). Brains were then cryoprotected in a solution of sucrose 30 % (w/v), at 4 °C, until they sank. Brains were frozen and sliced with a freezing microtome in 6 parallel coronal series of 40 μm (distance between slices in each parallel series: 240 μm). We employed the indirect ABC procedure for the detection of the neuronal marker Neu-N (1:100, MAB377 Millipore) in the first series, the reactive astroglial marker GFAP (1:500, Dako) in the second series and the microglial marker Iba1 (1:1000, Wako) in the third series. Briefly, sections were blocked with 2 % (v/v) Normal Goat Serum (NGS, Vector Laboratories) in PBS-Triton100 0.3 % (v/v) and endogenous peroxidases activity blocked with 1 % (v/v) hydrogen peroxide (H_2_O_2_) in PBS for 30 min at room temperature. Subsequently, sections were incubated for 30 min at room temperature in (*i*) primary antibody (at the concentration indicated above) in PBS with 0.3 % (v/v) Triton X-100 and 2 % (v/v) NGS, (*ii*) biotinylated secondary antibody in the same buffer, and (*iii*) avidin–biotin–peroxidase complex (ABC Elite kit Vector Laboratories) in PBS-Triton X-100 0.3 % (v/v). Sections were washed 3x10 min in PBS. The peroxidase activity was revealed with SIGMAFAST-DAB (3,3′-Diaminobenzidine tetrahydrochloride, Sigma-Aldrich) in PBS for 5 min. Sections were rinsed and mounted onto slides, cleared with Histoclear (Fisher Scientific) and coverslipped with Eukitt (Fluka). The fourth GFP-injected series was mounted onto slides and covered with Mowiol (Sigma-Aldrich) for fluorescence analysis.

### Image analysis

#### Determination of the volume of injection

Five coronal slices per GFP-injected hemisphere from bregma 1.5-mm levels, separated 240 μm, were photographed with a digital camera attached to a macrozoom microscope (Leica). The contours around GFP-expressing area and dorsal striatum were manually defined and the area was measured with ImageJ software (National Institute of Health, USA). Volume was calculated as area per distance between slices, according to the Cavalieri principle [30].

#### Determination of O.D. for GFAP and IBA1 stainings

Four coronal slices per mouse and hemisphere covering the striatum from bregma 1.5-mm levels were selected, and a region of interest of 670 × 897 μm^2^ in the middle of dorsal striatum was captured with a 10x objective using a digital camera attached to a microscope (Leica DMIRBE). The O.D. of the areas was measured with ImageJ, the mean density per hemisphere calculated and O.D. for GFAP and IBA1 of control hemispheres were subtracted from the injected hemisphere.

#### Determination of the neuronal density of the striatum

Cell density was calculated using an adaptation of the unbiased fractionator method [30]. Four coronal slices per mouse and hemisphere covering the striatum from bregma 1.5-mm levels were selected, and a region of interest of 447 × 598 μm^2^ in the middle of dorsal striatum was captured with a 15x objective using a digital camera attached to a microscope (Leica DMIRBE). A grid image leaving 16 squares of 35 × 35 μm^2^ was superimposed to the pictures and a person blinded to sample treatment counted the number of stained nuclei.

### Statistical analysis

Data were analysed StatPlus package for Excel (Microsoft) and IBM SPSS Statistics 22. To test the inflammatory response we calculated the difference of O.D. of the injected hemisphere versus the control hemisphere and performed a Student’s *t* test against no difference value (0). For neuronal density, we performed a paired Student’s *t* test of neuronal density in the injected hemisphere versus the control hemisphere. We analysed the neuronal density across contralateral hemispheres with an ANOVA, followed by post-hoc comparisons with the contralateral hemispheres of the PBS samples. To test repression, we calculated the percentage of mutant *HTT* (*mut HTT*) or the gene of interest (*Htt, Atn1, Atxn2, Tbp*) in the injected brain, with respect to the control hemisphere, and performed a one sample Student’s *t* test against the no repression value (100 %). To ensure a fair comparison between injected and contralateral hemispheres, only mice with <1 % ZF expression in the contralateral hemisphere, relative to the injected hemisphere, were used for statistical analyses (see Additional file 5 for the full dataset). To test the correlation between RNA levels of the different genes and ZF expression we applied a linear regression test. To test expression levels across different times post-injection we applied a one-way ANOVA. All significance values are set at *p* = 0.05.

## Results and discussion

### Design of the mouse host-matched ZF

Starting with the most effective lead construct from our previous paper (ZF11xHunt-KOX1 [14]; hereafter named ZF-KOX1), we designed a mouse host-matched ZF (mZF-KRAB) to reduce potential immunogenicity (see Additional file 1, for full annotated sequences). The design modifications in mZF-KRAB (Fig. 1a) are described more fully in Methods. Briefly, the changes include removing FLAG epitope tags and changing effector domains, such as nuclear localization signals and KRAB repressors, to mouse analogues. We also altered the zinc finger recognition helices to make them as close as possible to the mouse zif268 transcription factor sequence [22] (i.e., to reduce the potential for foreign epitopes). These changes were carried out within the constraint of retaining CAG-binding activity, using previous ZF ELISA experiments as a guide [24].

Overall, the original ZF-KOX1 had 260/509 non-mouse residues (51 %), whereas mZF-KRAB had 66/430 (15 %) (Fig. 1a). We thus reduced the non-wild-type mouse sequences in mZF-KRAB to a bare minimum, with all the remaining non-mouse sequences being essential for DNA binding functionality. In order to have a positive control for the toxicity of a foreign protein in mice, we chose GFP, which is derived from the jellyfish, *Aequorea victoria*. GFP toxicity after AAV delivery to the brain is well described in the literature [11-13] and was used as a benchmark to gauge how the ZF constructs fared by comparison.

### Host-matching reduces the microglial proliferation observed with ZF-KOX1 and GFP

Microglial cells are the main players in brain innate immune responses, so we tested whether various treatments activated microglia. We performed unilateral injections of recombinant adeno-associated viral constructs (rAAV2/1-ZF-KOX1, rAAV2/1-mZF-KRAB and rAAV2/1-GFP), or PBS, in the striata of wild-type (WT) mice (Fig. 1b). WT mice were used to avoid any confounding effects of the HD phenotype. To check the infection region covered by our injection procedure, we measured the volume covered by GFP fluorescence in GFP-injected samples (Fig. 1b). This revealed a consistent average of ~50 % infection, both at 4 and 6 weeks post-injection (Fig. 1c). No GFP fluorescence was apparent outside of the injected striatum. Since the number of viral copies and the vector amount used was the same for GFP, ZF-KOX1 and mZF-KRAB, we assume that the efficiency of transduction is similar in all groups.

**Fig. 2:**
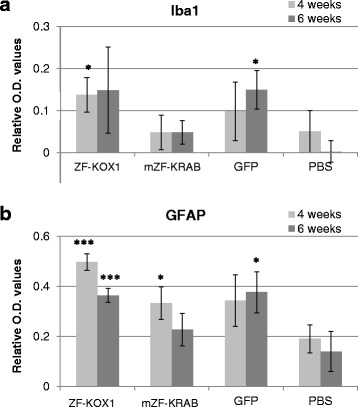
Relative O.D. values of the striatal samples immunostained for glial markers. Relative O.D values, representing inflammatory responses to various treatments, were calculated for the microglial marker IBA1 (**a**) and the reactive astroglial marker GFAP (**b**), at 4 and 6 weeks after injection. Unoptimized ZF-KOX1 treatment was compared to expression of a host optimized mZF-KRAB, GFP or a control PBS injection. Relative O.D. is calculated as the mean O.D. of four coronal slices, separated by 240 μm in the injected hemisphere, minus the average O.D. in the contralateral control hemisphere. Data are displayed as Relative O.D. ± S.E.M, *** *P* < 0.001, **P < 0.01, *P < 0.05

**Fig. 3:**
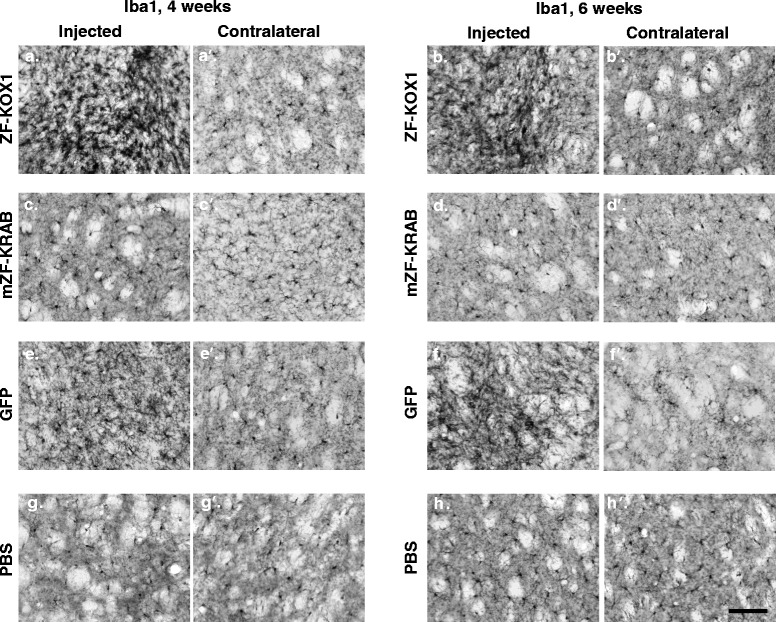
Microglial activation in the striatum after various treatments. Representative micrographs of IBA1 immunostained striatal coronal slices, for the control and injected hemispheres, for each treatment at 4 or 6 weeks. ZF-KOX1 samples displayed an apparent increase in Iba1 immunoreactivity in the injected hemispheres, at 4 and 6 weeks after treatment (**a**, **b**). This was not observed in the contralateral hemispheres (**a’**, **b’**). Hemispheres treated with mZF-KRAB showed similar levels of Iba1^+^ cells compared with their contralateral non-injected hemispheres (**c**, **c’**, **d**, **d’**). Certain GFP-treated samples showed a slight increase in Iba1 immunoreactivity 4 weeks after treatment (**e**). Iba1 immunoreactivity was significantly increased 6 weeks after GFP injections, compared with the contralateral hemispheres (**f**, **f’**). PBS-injected samples show similar Iba1 immunoreactivity between hemispheres at both time points (**g**, **g’**, **h**, **h’**). Scale bar: 100 μm

To measure microglial upregulation, we carried out immunostaining with a marker for the ionized calcium-binding adapter molecule 1 (Iba1). The tissues were then analysed by quantifying the O.D., following a previously published procedure [11]. A Student’s *t*-test, compared the O.D. value of the injected hemispheres against the background O.D. of the contralateral, non-injected hemisphere (Fig. 2a). This revealed that ZF-KOX1 and GFP provoked significant increases in microglia, at 4 and 6 weeks post-injection, respectively (c.f. Fig. 3a, f). For ZF-KOX1, the average values of O.D. at 6 weeks were similar to 4 weeks post-injection, but the increased variability in the samples prevented the result from reaching statistical significance. As some of these samples showed dense Iba1^+^ immunostaining, the conclusion is that ZF-KOX1 treatment results in a sustained inflammation at both 4 and 6 weeks after injection (Fig. 3a, b).

In contrast, mZF-KRAB and PBS injections did not significantly increase the amount of microglial staining (Fig. 3c, d, g, h). Only scattered enlarged microglial cells could be detected in the tissue, mainly surrounding the needle tract, in both the mZF-KRAB and PBS-injected hemispheres. Thus, the injection of the foreign proteins, ZF-KOX1 and GFP, induced a strong proliferation of microglial cells in WT mice, at different time points, which was not present in the case of the host-matched mZF-KRAB.

### mZF-KRAB provokes reduced and short-lived astroglial reactivity, relative to ZF-Kox1 and GFP

**Fig. 4:**
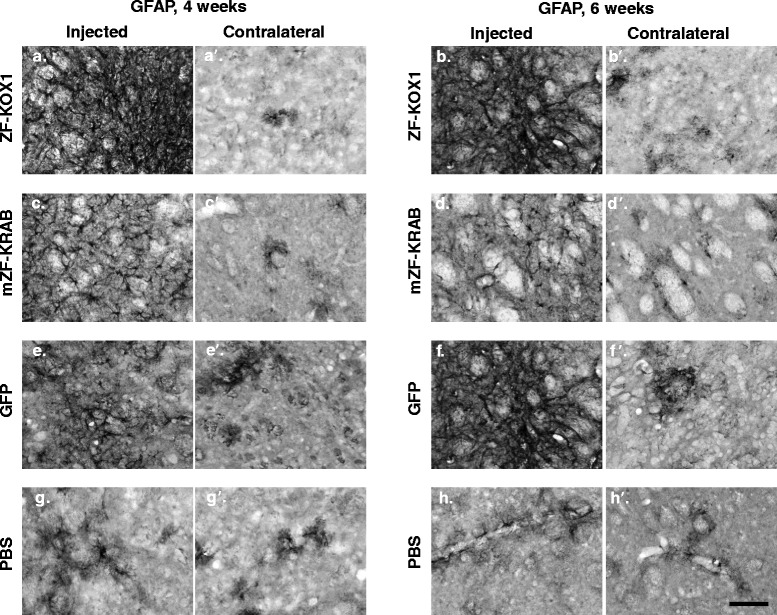
Astroglial activation in the striatum and cortex after various treatments. Representative micrographs of GFAP immunostained striatal coronal slices for the control and injected hemispheres, for each treatment at each time point. ZF-KOX1 samples displayed a strong and sustained increase in GFAP immunoreactivity in the injected hemispheres, 4 and 6 weeks after treatment (**a**, **b**). In contrast, mZF-KRAB treatment provoked a transient upregulation of GFAP immunoreactivity, which started to decline at 6 weeks post-injection (**c**, **d**). GFAP immunoreactivity after GFP injections followed the pattern of Iba1 staining, with a slight increase 4 weeks post-treatment and significant signal increase at 6 weeks post-treatment (**e**, **f**). Isolated, non-reactive astrocytes can be observed at PBS-injected samples and their contralateral hemispheres (**a’**, **b’**, **c’**, **d’**, **e’**, **d’**, **g**, **g’**, **h**, **h’**). Scale bar: 100 μm

We next checked if the treatments provoked an increase in reactive astroglia by immunostaining the mouse brain slices for glial fibrillary acidic protein (GFAP), and measuring O.D., as in the previous experiment. We compared the O.D. value of the injected hemispheres against the basal O.D. of the contralateral, non-injected hemisphere by means of a Student’s *t*-test (Fig. 2b). This revealed that GFAP was significantly upregulated in the injected hemispheres, in both time points, in the ZF-KOX1 samples (Fig. 4a, b). The mZF-KRAB samples showed less GFAP upregulation, and even this was mainly restricted to areas surrounding the needle tract (only significant at 4 weeks post injection) (Fig. 4c, d). In contrast, GFP caused a delayed reactivity, reaching a significant increase (with respect to the control hemisphere) at 6 weeks post-injection (Fig. 4f). The PBS control injection did not induce a significant increase in reactive astroglia (Fig. 4g, h). Isolated, non-reactive astrocytes were found in all contralateral, non-injected hemispheres (Fig. 4). Overall, ZF-KOX1 and GFP caused a persistent activation of astroglial cells in WT mice, whereas for mZF-KRAB and PBS this activation was weaker and was already reduced at week 6 post-injection.

### mZF-KRAB is less toxic than ZF-KOX1 or GFP, which both induce significant neuronal death

**Fig. 5:**
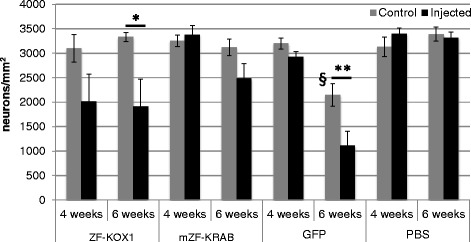
Quantification of striatal neuronal density after various treatments. Bar chart representing the estimated neuronal density in the striata of mice after the different treatments. Data are expressed as mean ± S.E.M. *p < 0.05; **p < 0.01; ^§^ *p* < 0.01 (^§^compares cell counts in the contralateral hemispheres of the 6-week GFP and PBS samples. GFP is the only treatment in this study where cell numbers are reduced in the contralateral, non-injected hemisphere)

**Fig. 6:**
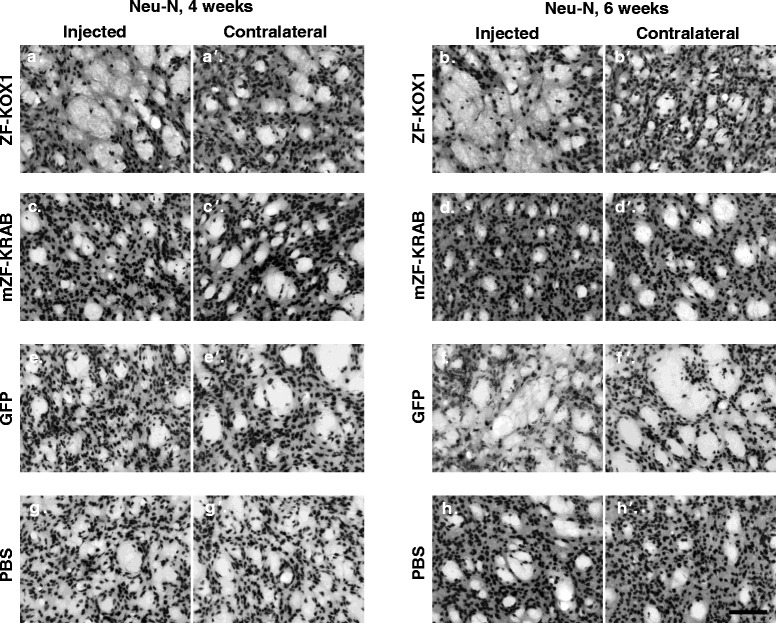
Visualising striatal neuronal density after various treatments. Representative micrographs of Neu-N immunostained striatal coronal slices for the control and injected hemisphere of each treatment at each time point. ZF-KOX1 toxicity is observed in areas of the injected striata that are devoid of marked neurons, 4 and 6 weeks after treatment (**a**, **b**), whereas the contralateral hemispheres (**a’**, **b’**) show neuronal densities similar to PBS injected (**g**, **h**) and untreated hemispheres (**g’**, **h’**). Conversely, mZF-KRAB treatment did not significantly affect neuronal density (**c**, **c’**, **d**, **d’**). Strikingly, GFP injections did not affect neuronal density at 4 weeks after treatment (**e**, **e**’), but caused a delayed strong toxic response that reduced neuronal density both in the injected (**f**) and the contralateral hemisphere (**f’**), 6 weeks post-injection. Scale bar: 100 μm

To verify whether the observed inflammatory responses were accompanied by neuronal loss, we used immunohistochemical detection of the neuronal marker NeuN. We first estimated the neuronal density in each hemisphere of the various treated animals (Fig. 5). A Student’s *t*-test between the injected and the non-injected hemispheres revealed that number of neurons showed a trend towards reduction by ZF-KOX1 by week 4 after injection (*p* = 0.08) (Fig. 5). This reduction reached significance by 6 weeks post-injection (*p* = 0.014). Moreover, for ZF-KOX1, we could observe extensive neuronal death in some samples after 6 weeks, resulting in no effective detection of NeuN in the area surrounding the injection (Fig. 6a, b). In fact, no Neu-N could be observed at all in some of the slices, which displayed only high background staining.

By contrast, mZF-KRAB did not affect neuronal density, either at 4 or 6 weeks after treatment (Fig. 6c, d). We observed a limited reduction in the number of neurons near the needle tract in two out of four animals at week 6 post-injection, but the numbers did not reach significance (*p* = 0.13). In general, most of the mZF-KRAB treated samples were similar to PBS injected hemispheres (Fig. 6g, h).

Finally, in agreement with the previous observations in Iba1^+^ and GFAP immunodetection, neuron density was not affected by GFP at 4 weeks post-treatment (Figs. 5, 6e), with only some areas showing scarcely-distributed neurons close to the needle tract. However, GFP significantly reduced neuronal density by week 6 post-injection (*p* = 0.018). The cytotoxicity of GFP was thus observed after a delay and was the strongest toxic effect observed in this study.

### ZF-KOX1 represses the *mut HTT* polyCAG target in vivo for up to 6 weeks

**Fig. 7:**
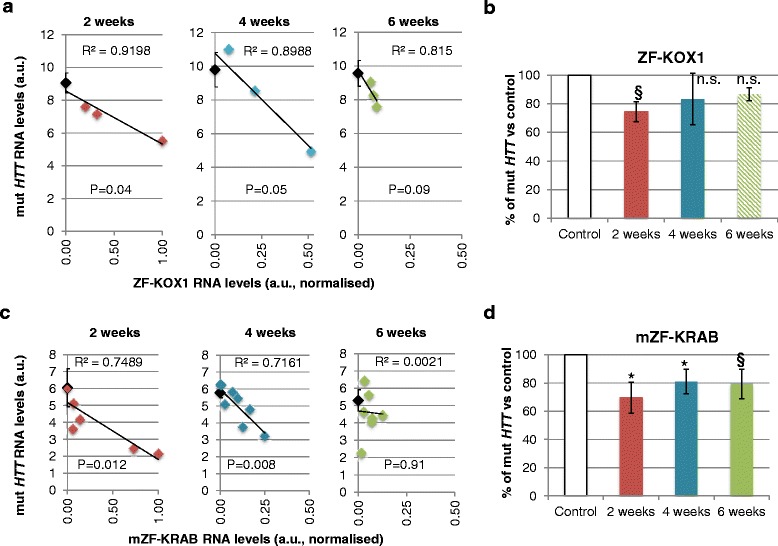
Mutant *huntingtin* gene expression analysis after treatment with ZFs. **a** Linear regression showing negative correlations of *mut HTT* RNA levels and ZF-KOX1 expression 2, 4, and 6 weeks after treatment, suggesting an effective repression of *mut HTT* by the treatment. Black diamonds show the mean *mut HTT* expression values (±1 S.E.M.) of the control hemispheres of each group. ZF-KOX1 expression levels are in arbitrary units (a.u), normalised to the maximum ZF-KOX1 qRT-PCR signal across all samples. **b** Percentage of *mut HTT* with respect to the average value in the control hemispheres, over the same period. The data show an average of ~25 % reduction of *mut HTT*, 2 weeks post-treatment (previously reported in [14]), with an individual mouse showing up to ~40 % reduction. The average percentage increases with time, but later values should be interpreted cautiously because of ZF-KOX1 expression leakage to the contralateral hemisphere, and because of the significant neuronal loss. **c** Linear regression analysis testing for negative correlations between *mut HTT* RNA levels and mZF-KRAB expression, at 2, 4 and 6 weeks after treatment. mZF-KRAB expression levels are in arbitrary units (a.u), normalised to the maximum mZF-KRAB qRT-PCR signal across all samples. **d** Percentage of *mut HTT* with respect to the average value in the control hemispheres over the same period. The columns show mean RNA expression levels; error bars: ±1 S.E.M. *p < 0.05; ^§^ *p* < =0.06

In previous work, we had determined that ZF-KOX1 functionally repressed its target in R6/2 HD model mice for 2-3 weeks after bilateral injection, during which time pathological clasping symptoms were virtually abolished [14]. We now explored whether repression was sustained for a longer period, up to 6 weeks. We therefore injected rAAV2/1-ZF-KOX1 into R6/2 mice only in one hemisphere and left the contralateral hemisphere uninjected, for baseline comparison. Taking samples 2, 4 and 6 weeks after injection, we analysed RNA levels via quantitative real-time PCR [14] (Fig. 7a).

To verify whether ZF-KOX1 was able to repress its target gene (*mut HTT*) in a dose-dependent manner, we carried out linear regression of the RNA levels of *mut HTT* versus ZF-KOX1, for each time point (Fig. 7a). There was a significant and negative correlation between these RNA levels 2 weeks after treatment (*p* = 0.04). There were also trends approaching significance at 4 and 6 weeks (*p* = 0.05, *p* = 0.09). These results indicate that, although there is variability in individual injected mice, higher expression of the ZF-KOX1 results in lower levels of *mut HTT*, which is consistent with previous results [14].

By comparing the *mut HTT* levels in the ZF-KOX1-injected hemisphere to the baseline in the uninjected hemisphere (Fig. 7b), we observed that ZF-KOX1 yielded a mean reduction in *mut HTT* of ~35 % after 2 weeks, which is in the therapeutic range [14]. Repression was maintained at ~20 % at 4 and 6 weeks after injection (Fig. 7b). However, ZF-KOX1 expression was accompanied by a significant cell loss (Figs. 5, 6). For this reason, we decided that ZF-KOX1 should not be used for any further experiments in mice and we did not extend this trial.

### mZF-KRAB represses the *mut HTT* polyCAG target in vivo

We next explored whether host-matched mZF-KRAB was a more suitable candidate for long-term repression of mutant huntingtin (*mut HTT*). The early-onset R6/2 phenotype is useful for phenotyping trials but, as a result of Ethical Review (see Methods), we refined the procedure for collecting ZF expression and repression data at 6 weeks, and switched to the later-onset HD mouse model, R6/1. Ethically, the use of R6/1 is preferred over R6/2 because the data can be collected before any HD symptoms are detected, maximising animal welfare. In fact, both R6/1 and R6/2 bear the same transgene, although the CAG repeats are slightly longer in the R6/2 mice we are using (~160 repeats versus ~120-130 in R6/1). This repeat number difference should not matter for quantifying repression, since we see comparable ZF repression in StHdh cells with 111 repeats [14]. Although R6/1 is thus a valid model for testing zinc finger repression, the repression data should not be formally compared to the results with R6/2, because of the different time-dependence of the HD phenotypes.

We injected rAAV2/1-mZF-KRAB into R6/1 mice and killed them at 2, 4, and 6 weeks post-injection, taking samples to analyse RNA levels via quantitative real-time PCR. mZF-KRAB RNA levels were negatively correlated with *mut HTT* at 2 and 4 weeks post-injection (*p* < 0.05) (Fig. 7c). This indicates that mZF-KRAB target repression is functioning. The linear correlation was lost by week 6 post-injection, although the majority of mice still had reduced *mut HTT* levels in the injected hemisphere, with respect to control levels. Interestingly, studies in mice have shown that transient repression of *mut HTT* with modified oligonucleotides can persist for 8 weeks after the treatment [17]. Therefore, it is likely that we are observing persistent repression of *mut HTT* at 6 weeks, which is no longer correlated to mZF-KRAB expression because of a trend towards a reduction in ZF expression over time, under the pCAG promoter [31]. It should be noted that KRAB domains lay down heterochromatin across genetic loci and thus cause strong long-term repression which can outlast their expression [21].

By comparing the *mut HTT* levels in the mZF-KRAB-injected hemisphere to the baseline in the uninjected hemisphere (Fig. 7d), we saw that repression was on average ~30 % at 2 weeks (*p* = 0.04) and stabilised around ~20 % at 4 and 6 weeks (*p* = 0.04, *p* = 0.05, respectively). Thus, mZF-KRAB is functionally active in repressing *mut HTT* at 6 weeks and this, together with its reduced apparent toxicity, makes this construct suitable for in vivo use.

### mZF-KRAB specifically represses *mut HTT* and not other CAG-containing genes

Since the mouse genome contains 7 potential polyQ expansion genes [14], we checked whether the transcriptional repression of *mut HTT* was specific or whether it affected some of these other polyCAG-targets. We tested the effects of ZF-KOX1 and mZF-KRAB on the expression of four of these genes (wild-type *WT Htt, Atn1*, *Atxn2, Tbp;* Table 1). The results show that the RNA levels of these genes were not negatively correlated with ZF expression, except, strikingly, in the case of *WT Htt* with ZF-KOX1, at all time points (Table 1). In particular, ZF-KOX1 significantly repressed ~10 % of mouse *WT Htt,* 2 and 4 weeks after treatment, in a dose-dependent manner (Table 1). Conversely, none of the gene expressions were correlated with mZF-KRAB at any point. Thus, at least under the conditions tested, mZF-KRAB appears to be specific against its target, which may also be contributing to its low apparent toxicity.

**Table 1:** Expression of mouse endogenous CAG-containing genes after treatment with ZF-KOX1 and mZF-KRAB

Treatment	Weeks post-injection	Linear regression	Htt (4,7) %	Linear regression	Atn1 (3,10) %	Linear regression	Atxn2 (6,10) %	Linear regression	Tbp (3,13) %
ZF-KOX1	2	***R*** ^**2**^ **= 0.97** ^**§**^	92.1 ± 0.4*	*R* ^2^ = 0.03	101.2 ± 13.8	*R* ^2^ = 0.09	95.5 ± 3.9	***R*** ^**2**^ **= 0.98** ^**§**^	94.5 ± 5.0
	4	***R*** ^**2**^ **= 0.99** ^**§**^	89.6 ± 2.4*	*R* ^2^ = 0.64	91.7 ± 13.7	*R* ^2^ = 0.57	96.1 ± 6.8	*R* ^2^ = 0.98	91.5 ± 1.7
	6	***R*** ^**2**^ = 0.99*	90.6 ± 9.6	*R* ^2^ = 0.09	106.3 ± 4.9	*R* ^2^ = 0.96	98.4 ± 2.9	*R* ^2^ = 0.65	98.1 ± 3.3
mZF-KRAB	2	*R* ^2^ = 0.53	98.3 ± 3.3	*R* ^2^ = 0.02	109.4 ± 10.6	*R* ^2^ = 0.00	103.3 ± 11.8	*R* ^2^ = 0.08	99.1 ± 7.9
	4	*R* ^2^ = 0.14	88.4 ± 6.7	*R* ^2^ = 0.19	88.6 ± 7.3	*R* ^2^ = 0.36	89.6 ± 5.4	*R* ^2^ = 0.43	90.5 ± 10.4
	6	*R* ^2^ = 0.43	92.5 ± 11.35	*R* ^2^ = 0.16	91.4 ± 6.5	*R* ^2^ = 0.00	96.7 ± 4.4	*R* ^2^ = 0.07	94.7 ± 4.8

The first number (in brackets after the name of the gene) represents the number of CAG repeats, the second the number of glutamines in the coding stretch (CAG + CAA). Values are given as the percentage expression of the gene of interest, with respect to the average values in the control hemispheres. The result of ZF-KOX1, 6 weeks post-injection, should be taken with caution, since at this time point there was a significant neuronal loss and leakage of the vector to the contralateral hemisphere. In bold: ^§^ *P* < 0.1; *P < 0.05. Atn1: atrophin 1; Atxn2: ataxin 2; *Htt*: huntingtin (mouse); Tbp: TATA binding protein

### A non-viral promoter for long-term ZF expression and functional *mut HTT* repression

Up to this point, the ZFs used in this study had been expressed from a very strong pCAG promoter (CMV early enhancer element and chicken β-actin promoter) with the Woodchuck hepatitis virus postranscriptional regulatory element (WPRE). Although this construct achieved strong repression of *mut HTT*, there was a trend to loss of transgene expression over 6 weeks. Since this was not attributable to cell loss in the case of mZF-KRAB, it was possible that promoter silencing was occurring. Indeed, it has been found that the pCAG promoter is methylated and may lose transgene expression over time [31]. Consequently, we explored whether a non-viral promoter might be better for maintaining transgene expression.

As an alternative to pCAG, we selected the 1.8 kb rat neuron specific enolase promoter (pNSE), because this was shown to give persistent expression that is several 100-fold greater than the short CMV promoter in rodent brains [32, 33]. In combination with a WPRE, pNSE gave strong stable luciferase expression in the rat striatum, even at 15 months post injection [34].

**Fig. 8:**
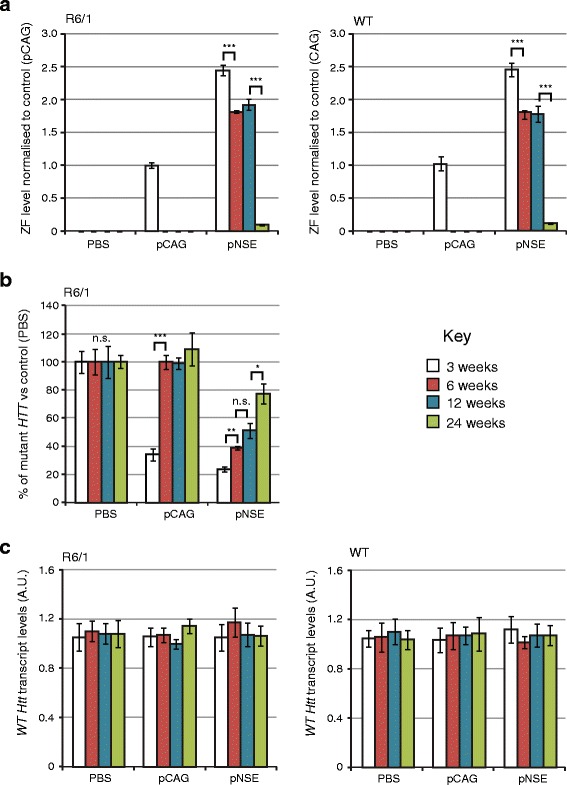
Long-term effects of bilateral intraventricular injection of AAV expressing mZF-KRAB under pCAG or pNSE promoters. **a** Zinc finger expression over time. mZF-KRAB transcript levels from whole brains were assayed by qRT-PCR at 3, 6, 12 and 24 weeks after viral (or PBS control) injections, in WT or R6/1 neonates. **b** Zinc finger repression of mutant Huntingtin in R6/1 mice. *mut HTT* (exon 1) expression levels in the whole brain samples from the various treatments were compared to transcript levels in PBS controls, by qRT-PCR. **c** Verification of lack of cross-reactivity of mZF-KRAB with short WT *Htt* alleles. WT *Htt* (exon 1) expression levels were quantified in the same treatment samples as above. Housekeeping genes and other control data are shown in Additional file 5. Error bars are S.E.M (*n* = 3). ** *p* < 0.01, *** *p* < 0.001, n.s. = not significant

The pNSE promoter was synthesized chemically and vectorised to express mZF-KRAB-WPRE, using rAAV2/1. To set up long term ZF expression and mutant *HTT* repression experiments, we carried out bilateral intraventricular injection on neonatal R6/1 and WT mice, with the maximum volume of AAV possible (4 μl, ~10^10^ virions). Whole brains were harvested after 3, 6, 12 and 24 weeks and analysed by qPCR to determine ZF expression and mutant *HTT* repression, while PBS and the previous pCAG promoter construct were used as controls. We found that whereas pCAG promoter activity was lost by 6 weeks, the pNSE promoters had relatively stable expression up to 12 weeks (74-78 % of maximum) and ZF was still detectable after 24 weeks (4-5 % of maximum pNSE levels and 10-12 % of maximum pCAG levels in WT and R6/1) (Fig. 8a). It should be noted that the detection of ZF is on a relative scale and that even ~5-10 % of maximum may still indicate an absolute concentration of ZF that is still functionally active; this can only be determined by examining the repression of target mutant *HTT* (Fig. 8b).

When looking at repression of mutant *HTT*, pCAG-driven repression was only detected at 3 weeks and was lost by 6 weeks (Fig. 8b), matching the complete loss of detectable ZF expression from this promoter. By contrast, pNSE-driven repression was maintained over the entire 24-week period, albeit with a trend to reduction over time (Fig. 8b). At 3, 6, 12 and 24 weeks, respectively, this resulted in 77 %, 61 %, 48 % and 23 % repression of mutant *HTT* in whole brain samples. Notably, pNSE-ZF still repressed mutant *HTT* levels by nearly a quarter after 24 weeks, which is close to levels previously found to be in the therapeutic range (Fig. 6a of Ref. [14]). As expected, the short WT *Htt* allele was unaffected in all samples, indicating a lack of off-target activity (Fig. 8c). Overall, the results show that combining our host-adapted mZF-KRAB design with the pNSE promoter allows targeted *mut HTT* repression in the whole brain, for an extended 6 month period that is much longer than the 3 weeks reported previously [14].

## Conclusions

The humanization of biologics, such as engineered antibodies, is long established as a way of reducing immunoreactivity in therapy [35, 36]. Adapting synthetic gene therapy constructs for their hosts is relatively more challenging because they can have more components, and longer functional regions have to be considered. For example, multiple zinc finger binding helices usually require several non-wild-type amino acids to bind desired target sequences (Fig. 1a). Nonetheless, these differences can and should be minimized.

The first aim of this study was to replace all effector domains with host homologs and to reengineer the functional DNA-binding domain to retain its designed activity. The latter required using the fewest possible amino acid changes, relative to the host organism protein scaffold (in this case, the mouse zinc finger domain, Zif268). The design changes we introduced were sufficient to reduce neuronal cell loss, making the gene therapy construct less toxic than even a GFP expression vector; in fact, the mouse host-adapted construct behaved more like a control PBS injection.

The reduction of inflammatory responses following host-matching of the ZF construct suggests an immune response against non-self proteins as the most likely explanation for the detected medium-term toxicity. However, when host-matching, we made several changes, including differences in the NLS, ZF DNA-binding helices, and KRAB repressor domain. It is therefore possible that certain individual elements contribute to reduced immunogenicity or toxicity, rather than broad host-matching; without further testing it is difficult to extend the conclusions outside of this system and future work is needed to establish whether such host-matching is a generic strategy. Furthermore, although the host-matching changes aimed at conserving function (i.e., DNA binding to poly-CAG and KRAB repression), it is possible that these changes introduced subtle differences which contributed to lower toxicity. For example, although the mouse KRAB domain was chosen to have similar transrepressing activity to KOX1 [23], it may have had altered activity which resulted in lower off-target effects. Similarly, mZF-KRAB was more specific against off-target short poly-CAG genes (Table 1), which may also have contributed to its apparent lower toxicity. Arguing against this is our previous observation of the lack of apparent toxicity of ZF-KOX1, both in cell culture and in short term in vivo assays. Indeed, we were able to generate stable cell lines expressing ZF-KOX1 in vitro, and a cell viability assay showed no toxic effects [14]. Therefore the strong inflammatory responses seen with ZF-KOX1, which were absent with mZF-KRAB, remain a likely reason behind the improved tolerance of the latter in vivo.

The apparent success of mouse host-adaptation raises the question of what construct should be made for an eventual human therapy. Based on the results presented here, we would suggest that the effector domains (i.e., NLS, zinc finger scaffold, KRAB domain) should all be human. The engineered changes required for function (e.g., ZF linkers, ZF DNA recognition helices) should all be kept to a minimum and should be as close as possible to the corresponding human scaffold sequences. In principle, epitope scanning [37] can also help to guide the final choice of amino acid design changes, with the aim being to reduce potential epitopes in the remaining non-human regions.

The second aim of this study was to explore whether the loss of activity of the pCAG promoter could be bypassed by using a non-viral promoter-enhancer, and whether this would maintain measurable levels of *mut HTT* repression in whole brain. Endogenous promoters have been developed for gene therapy [38] but, to our knowledge, no-one has tested a promoter for long-term expression of a transgene transcription factor. Our results show that the 1.8 kb rat neuron specific enolase Eno2 promoter (pNSE [34]) is active for this purpose in the whole brain. Although maximal mutant *HTT* repression of 77 % was observed after 3 weeks, 23 % repression was still observed after ~6 months, which is a striking improvement upon previous pCAG (CMV-enhanced) constructs.

Despite these improvements, we still observed a slow reduction in ZF expression over time with our final construct, rAAV2/1:pNSE-mZF-KRAB-WPRE. The cause is not immediately apparent, and there appears to be a drop between 12 and 24 weeks (expression is relatively stable between 6 and 12 weeks; Fig. 8a). Promoter shutdown and cell loss are possibilities, although the former goes against previous reports [34], and the latter is unlikely because it would likely be accompanied with a severe phenotype, which we did not observe. An alternative is that viral copy number per cell is reduced over time: the mice are still growing over this period and it is possible that we are seeing a dilution of the non-replicative AAV vector as cells divide. If this is true, then expression might be maintained from those cells that received a higher initial dose. It should be noted that in the final experiments (Fig. 8) we have used larger AAV doses than were previously possible (because of previous limitations in viral titre). With these doses, we have observed the largest TF repression effect seen thus far (up to 77 % in whole brain). In the future, by using even more concentrated viral preparations, it may be possible to increase the AAV dose-per-cell, and the percentage of the brain that is infected, thus potentially increasing both the treatment effect and period. Nonetheless, the current results - showing that we have reduced mutant Huntingtin levels by around a quarter in the whole brain after six months - demonstrate that we have achieved our main goal of long term repression.

Overall, developing constructs that function in the long term is an important aim of gene therapy and the combination of host optimization and the use of a non-viral promoter in this study is a major step forward.

## Additional files

Additional file 1: DNA and protein sequences used. (DOCX 162 kb) Additional file 2: Summary of mice injected with ZF-KOX1, mZF-KRAB, GFP or PBS. (DOCX 48 kb) Additional file 3: Primers used in qRT-PCR analyses. (DOCX 100 kb) Additional file 4: Identification of reference genes for qPCR from whole brain of the R6/1 and WT mice. (DOCX 51 kb) Additional file 5: Raw data for the expression of ZF-KOX1 and mZF-KRAB. (XLSX 12 kb)

## Abbreviations

AAV9: Adeno-associated virus serotype 9
AIDS: Acquired immune deficiency syndrome
CCR5: Chemokine receptor type 5
CD4: Cluster of differentiation 4
CMV: Cytomegalovirus
*CRISPR*: Clustered regularly interspaced short palindromic repeats
*ELI*SA: Enzyme-linked immunosorbent assay
*GFP*: Green fluorescent protein
*HIV*: Human immunodeficiency virus
HTT: Huntingtin
KRAB: Krüppel associated box
mut: mutant
NLS: Nuclear localization signal
NSE: Neuron specific enolase
pCAG: CMV early enhancer element and chicken β-actin promoter
PCR: Polymerase chain reaction
qRT-PCR: Quantitative reverse transcription polymerase chain reaction
WPRE: Woodchuck hepatitis virus postranscriptional regulatory element
WT: Wild-type
ZF: Zinc finger
