# Supplementary material for: Deimmunization for gene therapy: host matching of synthetic zinc finger constructs enables long-term mutant Huntingtin repression in mice
Source: Mol Neurodegener. 2016 Sep 6;11(1):64. doi: 10.1186/s13024-016-0128-x (PMC5013590; doi:10.1186/s13024-016-0128-x)
Supplement: Additional file 1: — DNA and protein sequences used. (DOCX 162 kb) [file 13024_2016_128_MOESM1_ESM.docx]

**Additional file 1.**

**1. The DNA sequences of ZF used in this study**

The following coding sequences were cloned into the rAAV2/1 vector so that they would be expressed under a strong CAG promoter (CMV early enhancer element and the chicken β-actin promoter) and WPRE, as described previously (*Proc Natl Acad Sci USA* **109** (45): E3136-E3145 (2012) doi:10.1073/pnas.1206506109).

---

**Key**

ATG=start codon

3XFLAG-TAG

**NLS: nuclear localisation signal**

**KRAB repressor domain**

TAA/TGA=stop codon

---

**Original design (PNAS 109: E3136-45, 2012):**

>ZF11xHunt-KoxI_DNA=ZF-KOX1

ATGGCCgactacaaagaccatgacggtgattataaagatcatgacatcgactacaaagacgatgacgacaag**CCGAAGAAAAAACGTAAAGTG**ACCGGGGCCGAGCGCCCCTTCCAGTGCCGCATTTGTATGCGCAACTTTAGCCAGCGCGCGACCCTGCAGCGCCATACCAAAATTCACACCGGATCCGAACGGCCGTTTCAGTGCCGTATTTGCATGCGTAATTTTAGCCAGCGTGCGACCCTGCAGCGCCATATTCGTACCCATACCGGTGAAAAACCGTTTGCCTGCGATATTTGTGGCCGTAAATTTGCCCAGCGCGCGACCCTGCAGCGCCATACCAAAATTCATACCGGTTCTGAACGGCCGTTTCAGTGCAGGATTTGCATGCGTAATTTTTCCCAGCGCGCGACCCTGCAGCGCCATATTCGCACCCATACTGGTGAAAAACCGTTTGCCTGCGATATTTGCGGTCGTAAATTTGCGCAGCGTGCTACCTTACAGCGCCATACCAAAATTCATCTGCGCCAGAAAGATGGTGGCGGCGGCTCAGGTGGCGGCGGTAGTGGTGGCGGCGGCTCACAACTAGTCGGTACCGCCGAGCGCCCCTTCCAGTGCCGCATTTGTATGCGCAACTTTAGCCAGCGCGCGACCCTGCAGCGTCATATTCGCACCCATACCGGTGAAAAACCGTTTGCGTGCGATATTTGCGGTCGTAAATTTGCGCAGCGTGCGACCCTGCAGCGCCATACCAAAATTCACACCGGATCCGAACGGCCGTTTCAGTGCCGTATTTGCATGCGTAATTTTAGCCAGCGTGCGACCCTGCAGCGCCATATTCGTACCCATACCGGTGAAAAACCGTTTGCCTGCGATATTTGTGGCCGTAAATTTGCCCAGCGCGCGACCCTGCAGCGCCATACCAAAATTCATACCGGTTCTGAACGGCCGTTTCAGTGCAGGATTTGCATGCGTAATTTTTCCCAGCGCGCGACCCTGCAGCGCCATATTCGCACCCATACTGGTGAAAAACCGTTTGCCTGCGATATTTGCGGTCGTAAATTTGCGCAGCGTGCTACCTTACAGCGCCATACCAAAATTCATCTGCGCCAGAAAGATGGTGGCGGCGGCTCAGGTGGCGGCGGTAGTGGTGGCGGCGGCTCACAACTAGTC**TCTAGtttgtctcctcagcactctgctgtcactcaaggaagtatcatcaagaacaaggagggcatggatgctaagtcactaactgcctggtcccggacactggtgaccttcaaggatgtatttgtggacttcaccagggaggagtggaagctgctggacactgctcagcagatcgtgtacagaaatgtgatgctggagaactataagaacctggtttccttgggttatcagcttactaagccagatgtgatcctccggttggagaagggagaagagccctggctggtggagagagaaattcaccaagagacccatcctgattcagagactgcatttgaaatcaaatcatcagttTAA**

**Mouse host-matched design:**

**NLS from mouse primase p58 NLS (RIRKKLR)**

**KRAB from Zfp87**

>mZF-KRAB

ATGGGC**CGCATTAGAAAGAAACTCAGA**CTCGCAGAAAGACCTTACGCCTGTCCTGTGGAATCCTGTGATAGACGGTTCAGCCAGAGCGGGGATCTGACAAGGCACATCAGAATTCATACTGGGTCCCAGAAGCCCTTCCAGTGCCGGATCTGTATGCGCAACTTTAGCCAGTCCGGAGACCTCACCCGACACATCCGAACCCATACAGGGGAGAAGCCTTTCGCCTGCGACATTTGTGGTCGGAAATTTGCTCAGAGCGGCGATAGGAAGAGACACACAAAAATCCATACTGGCTCCCAGAAGCCATTCCAGTGCCGAATTTGTATGAGGAATTTTTCTCAGAGTGGCGACCTGACTCGACACATCAGGACTCATACCGGCGAAAAGCCCTTCGCATGCGACATTTGTGGAAGGAAATTTGCCCAGTCTGGGGATCGGAAGCGCCACACCAAAATCCATCTCAGACAGAAGGACGGAGGAGGAGGTTCCGGAGGAGGAGGTAGTGGCGGAGGGGGTTCACAGAAGCCTTTCCAGTGCAGAATCTGTATGCGGAACTTTTCACAGAGCGGAGATCTGACCAGACACATCCGGACACATACTGGGGAGAAGCCATTCGCTTGCGACATTTGTGGTAGGAAATTTGCACAGTCTGGCGATCGAAAGAGGCACACCAAAATCCATACAGGAAGTCAGAAACCTTTCCAGTGCCGCATTTGTATGCGAAATTTTTCCCAGTCTGGTGACCTGACACGCCATATTCGAACCCATACAGGGGAAAAACCTTTCGCCTGTGACATTTGTGGAAGAAAATTTGCTCAGAGCGGGGATAGAAAGCGGCACACTAAAATCCATACCGGCTCTCAGAAACCATTCCAGTGCCGGATTTGTATGCGCAACTTTAGTCAGTCAGGCGACCTGACCAGACACATCAGAACTCACACCGGAGAGAAACCCTTCGCATGTGATATCTGTGGTCGGAAATTCGCCCAGAGCGGCGATCGCAAGCGACACACTAAAATCCACCTCCGCCAGAAGGACGGCGGAGGATCCGGAGGGGGTGGGTCC**GAAGAGATGCTCAGTTTTAGAGATGTCGCTATTGACTTTTCAGCCGAGGAATGGGAGTGCCTGGAACCTGCCCAGTGGAACCTGTACAGGGACGTGATGCTGGAGAATTATAGCCACCTGGTCTTCCTGGGCCTCGCCTCCTGCAAGCCCTACCTCGTGACCTTTCTCGAACAGAGGCAGGAGCCAAGCGTCGTCAAGAGACCAGCAGCAGCAACCGTCCATCCA**TGA

**2. The protein sequences of ZF used in this study**

**Key:**

XXX = non-mouse sequences

M=start methionine

3XFLAG-TAG

**NLS: nuclear localisation signal**

Zinc finger sequence (from mouse zif268)

ZF DNA recognition helices underlined

**ZF linker sequences (e.g. TGEKP) in bold**

**Kox-1 or ZF87 KRAB repressor domain**

*=stop codon

**Original design (260/509 non-mouse residues; 51 %):**

>ZF11xHunt-KoxI_protein=ZF-KOX1 (binds 5'-polyGCA-3' dsDNA)

MADYKDHDGDYKDHDIDYKDDDDK **PKKKRKV TGAERP**

FQCRICMRNFS QRATLQR HTKIH **TGSERP**

FQCRICMRNFS QRATLQR HIRTH **TGEKP**

FACDICGRKFA QRATLQR HTKIH **TGSERP**

FQCRICMRNFS QRATLQR HIRTH **TGEKP**

FACDICGRKFA QRATLQR HTKIH **LRQKDGGGGSGGGGSGGGGSQLVGTAERP**

FQCRICMRNFS QRATLQR HIRTH **TGEKP**

FACDICGRKFA QRATLQR HTKIH **TGSERP**

FQCRICMRNFS QRATLQR HIRTH **TGEKP**

FACDICGRKFA QRATLQR HTKIH **TGSERP**

FQCRICMRNFS QRATLQR HIRTH **TGEKP**

FACDICGRKFA QRATLQR HTKIH **LRQKDGGGGSGGGGSGGGGSQLV**

**SSLSPQHSAVTQGSIIKNKEGMDAKSLTAWSRTLVTFKDVFVDFTREEWKLLDTAQQIVYRNVMLENYKNLVSLGYQLTKPDVILRLEKGEEPWLVEREIHQETHPDSETAFEIKSSV***

**Mouse host-matched design (66/430 non-mouse residues; 15 %):**

>mZF-KRAB (binds 5'-polyGCA-3' dsDNA)

MG **RIRKKLR** LA **ERP**

YACPVESCDRRFS QSGDLTR HIRIH **TGSQKP**

FQCRICMRNFS QSGDLTR HIRTH **TGEKP**

FACDICGRKFA QSGDRKR HTKIH **TGSQKP**

FQCRICMRNFS QSGDLTR HIRTH **TGEKP**

FACDICGRKFA QSGDRKR HTKIH **LRQKDGGGGSGGGGSGGGGSQKP**

FQCRICMRNFS QSGDLTR HIRTH **TGEKP**

FACDICGRKFA QSGDRKR HTKIH **TGSQKP**

FQCRICMRNFS QSGDLTR HIRTH **TGEKP**

FACDICGRKFA QSGDRKR HTKIH **TGSQKP**

FQCRICMRNFS QSGDLTR HIRTH **TGEKP**

FACDICGRKFA QSGDRKR HTKIH **LRQKDGGGSGGGGS**

**EEMLSFRDVAIDFSAEEWECL EPAQWNLYRDVMLENYSHLVFLGLASCKPYLVTFLEQRQEPSVVKRPAAATVHP***

**Wild-type mouse 3-zinc finger scaffold - Zif268**

>mouse Zif268 (binds 5'-GCG-KGG-GCG-3' dsDNA)

M**AERP**

YACPVESCDRRFS RSDELTR HIRIH **TGQKP**

FQCRICMRNFS RSDHLTT HIRTH **TGEKP**

FACDICGRKFA RSDERKR HTKIH **LRQKD**

**3. The DNA sequence of the non-viral pNSE promoter used in this study**

The promoter was cloned into our AAV-NheI-pCAG-NheI-ZF-WPRE vector using NheI sites, by the Unitat de Producció de Vectors (UPV), Barcelona:

KEY:

gctagc= NheI

gatagc= cryptic NheI site (mutated to delete NheI)

TSS in capitals.

**pNSE**

>rat_eno2_NSE_promoter_plus_exon_1_1808bp

gctagcctcctctgctcgcccaatccttccaaccccctatggtggtatggctgacacagaaaatgtctgctcctgtatgggacatttgcccctcttctccaaatataagacaggatgaggcctagcttttgctgctccaaagttttaaaagaacacattgcacggcatttagggactctaaagggtggaggaggaatgagggaattgcatcatgccaaggctggtcctcatccatcactgcttccagggcccagagtggcttccaggaggtattcttacaaaggaagcccgatctgtagctaacactcagagcccattttcctgcgttaacccctcccgacctcatatacaggagtaacatgatcagtgacctgggggagctggccaaactgcgggacctgcccaagctgagggccttggtgctgctggacaacccctgtgccgatgagactgactaccgccaggaggccctggtgcagatggcacacctagagcgcctagacaaagagtactatgaggacgaggaccgggcagaagctgaggagatccgacagaggctgaaggaggaacaggagcaagaactcgacccggaccaagacatggaaccgtacctcccgccaacttagtggcacctctagcctgcagggacagtaaaggtgatggcaggaaggcagcccccggaggtcaaaggctgggcacgcgggaggagaggccagagtcagaggctgcgggtatctcagatatgaaggaaagatgagagaggctcaggaagaggtaagaaaagacacaagagaccagagaagggagaagaattagagagggaggcagaggaccgctgtctctacagacatagctggtagagactgggaggaagggatgaaccctgagcgcatgaagggaaggaggtggctggtggtatatggaggatgtagctgggccagggaaaagatcctgcactaaaaatctgaagctaaaaataacaggacacggggtggagaggcgaaaggagggcagagtgaggcagagagactgagaggcctggggatgtgggcattccggtagggcacacagttcacttgtcttctctttttccaggaggccaaagatgctgacctcaagaactcataataccccagtggggaccaccgcattcatagccctgttacaagaagtgggagatgttcctttttgtcccagactggaaatccgttacatcccgaggctcaggttctgtggtggtcatctctgtgtggcttgttctgtgggcctacctaaagtcctaagcacagctctcaagcagatccgaggcgactaagatgctagtaggggttgtctggagagaagagccgaggaggtgggctgtgatggatcagttcagctttcaaataaaaaggcgtttttatattctgtgtcgagttcgtgaacccctgtggtgggcttctccatctgtctgggttagtacctgccactatactggaataaggggacgcctgcttccctcgagttggctggacaaggttatgagcatccgtgtacttatggggttgccagcttggtcctggatcgcccgggcccttcccccacccgttcggttccccaccaccacccgcgctcgtacgtgcgtctccgcctgcagctcttgactcatcggggccccccgggtcacatgcgctcgctcggctctataggcgccgccccctgcccac**CCCCCGCCCGCGCTGGGAGCCGCAGCCGCCGCCACTCCTGCTCTCTCTGCGCCGCCGCCGTCACCACCGCCACCGCCACCGGCTGAGTCTGCAGTC**gctagc

4. Full AAV vector sequences

>pCAG-mZF-KRAB-mouse-QSGD-WPRE-B11 complete sequence 8277 bp

CCCGGGCAAAGCCCGGGCGTCGGGCGACCTTTGGTCGCCCGGCCTCAGTGAGCGAGCGAGCGCGCAGAGAGGGAGTGGCCAACTCCATCACTAGGGGTTCCTTGTAGTTAATGATTAACCCGCCATGCTACTTATCTACTCGACATTGATTATTGACTAGTTATTAATAGTAATCAATTACGGGGTCATTAGTTCATAGCCCATATATGGAGTTCCGCGTTACATAACTTACGGTAAATGGCCCGCCTGGCTGACCGCCCAACGACCCCCGCCCATTGACGTCAATAATGACGTATGTTCCCATAGTAACGCCAATAGGGACTTTCCATTGACGTCAATGGGTGGAGTATTTACGGTAAACTGCCCACTTGGCAGTACATCAAGTGTATCATATGCCAAGTACGCCCCCTATTGACGTCAATGACGGTAAATGGCCCGCCTGGCATTATGCCCAGTACATGACCTTATGGGACTTTCCTACTTGGCAGTACATCTACGTATTAGTCATCGCTATTACCATGGTCGAGGTGAGCCCCACGTTCTGCTTCACTCTCCCCATCTCCCCCCCCTCCCCACCCCCAATTTTGTATTTATTTATTTTTTAATTATTTTGTGCAGCGATGGGGGCGGGGGGGGGGGGGGGGCGCGCGCCAGGCGGGGCGGGGCGGGGCGAGGGGCGGGGCGGGGCGAGGCGGAGAGGTGCGGCGGCAGCCAATCAGAGCGGCGCGCTCCGAAAGTTTCCTTTTATGGCGAGGCGGCGGCGGCGGCGGCCCTATAAAAAGCGAAGCGCGCGGCGGGCGGGAGTCGCTGCGTTGCCTTCGCCCCGTGCCCCGCTCCGCGCCGCCTCGCGCCGCCCGCCCCGGCTCTGACTGACCGCGTTACTCCCACAGGTGAGCGGGCGGGACGGCCCTTCTCCTCCGGGCTGTAATTAGCGCTTGGTTTAATGACGGCTTGTTTCTTTTCTGTGGCTGCGTGAAAGCCTTGAGGGGCTCCGGGAGGGCCCTTTGTGCGGGGGGAGCGGCTCGGGGGGTGCGTGCGTGTGTGTGTGCGTGGGGAGCGCCGCGTGCGGCTCCGCGCTGCCCGGCGGCTGTGAGCGCTGCGGGCGCGGCGCGGGGCTTTGTGCGCTCCGCAGTGTGCGCGAGGGGAGCGCGGCCGGGGGCGGTGCCCCGCGGTGCGGGGGGCTGCGAGGGGAACAAAGGCTGCGTGCGGGGTGTGTGCGTGGGGGGGTGAGCAGGGGGTGTGGGCGCGTCGGTCGGGCTGCAACCCCCCCTGCACCCCCCTCCCCGAGTTGCTGAGCACGGCCCGGCTTCGGGTGCGGGGCTCCGTACGGGGCGTGGCGCGGGGCTCGCCGTGCCGGGCGGGGGGTGGCGGCAGGTGGGGGTGCCGGGCGGGGCGGGGCCGCCTCGGGCCGGGGAGGGCTCGGGGGAGGGGCGCGGCGGCCCCCGGAGCGCCGGCGGCTGTCGAGGCGCGGCGAGCCGCAGCCATTGCCTTTTATGGTAATCGTGCGAGAGGGCGCAGGGACTTCCTTTGTCCCAAATCTGTGCGGAGCCGAAATCTGGGAGGCGCCGCCGCACCCCCTCTAGCGGGCGCGGGGCGAAGCGGTGCGGCGCCGGCAGGAAGGAAATGGGCGGGGAGGGCCTTCGTGCGTCGCCGCGCCGCCGTCCCCTTCTCCCTCTCCAGCCTCGGGGCTGTCCGCGGGGGGACGGCTGCCTTCGGGGGGGACGGGGCAGGGCGGGGTTCGGCTTCTGGCGTGTGACCGGCGGCTCTAGAGCCTCTGCTAACCATGTTCATGCCTTCTTCTTTTTCCTACAGCTCCTGGGCAACGTGCTGGTTATTGTGCTGTCTCATCATTTTGGCAAAGAATTGATTAATTCGAGCGAACGCGTCGAGTCGCTCGGTACGATTTAAATTGaattggcctcgagcgcaagcttgagctagcctcgagaccATGGGCCGCATTAGAAAGAAACTCAGACTCGCAGAAAGACCTTACGCCTGTCCTGTGGAATCCTGTGATAGACGGTTCAGCCAGAGCGGGGATCTGACAAGGCACATCAGAATTCATACTGGGTCCCAGAAGCCCTTCCAGTGCCGGATCTGTATGCGCAACTTTAGCCAGTCCGGAGACCTCACCCGACACATCCGAACCCATACAGGGGAGAAGCCTTTCGCCTGCGACATTTGTGGTCGGAAATTTGCTCAGAGCGGCGATAGGAAGAGACACACAAAAATCCATACTGGCTCCCAGAAGCCATTCCAGTGCCGAATTTGTATGAGGAATTTTTCTCAGAGTGGCGACCTGACTCGACACATCAGGACTCATACCGGCGAAAAGCCCTTCGCATGCGACATTTGTGGAAGGAAATTTGCCCAGTCTGGGGATCGGAAGCGCCACACCAAAATCCATCTCAGACAGAAGGACGGAGGAGGAGGTTCCGGAGGAGGAGGTAGTGGCGGAGGGGGTTCACAGAAGCCTTTCCAGTGCAGAATCTGTATGCGGAACTTTTCACAGAGCGGAGATCTGACCAGACACATCCGGACACATACTGGGGAGAAGCCATTCGCTTGCGACATTTGTGGTAGGAAATTTGCACAGTCTGGCGATCGAAAGAGGCACACCAAAATCCATACAGGAAGTCAGAAACCTTTCCAGTGCCGCATTTGTATGCGAAATTTTTCCCAGTCTGGTGACCTGACACGCCATATTCGAACCCATACAGGGGAAAAACCTTTCGCCTGTGACATTTGTGGAAGAAAATTTGCTCAGAGCGGGGATAGAAAGCGGCACACTAAAATCCATACCGGCTCTCAGAAACCATTCCAGTGCCGGATTTGTATGCGCAACTTTAGTCAGTCAGGCGACCTGACCAGACACATCAGAACTCACACCGGAGAGAAACCCTTCGCATGTGATATCTGTGGTCGGAAATTCGCCCAGAGCGGCGATCGCAAGCGACACACTAAAATCCACCTCCGCCAGAAGGACGGCGGAggatccggagggggtgggtccgaagagatgctcagttttagagatgtcgctattgacttttcagccgaggaatgggagtgcctggaacctgcccagtggaacctgtacagggacgtgatgctggagaattatagccacctggtcttcctgggcctcgcctcctgcaagccctacctcgtgacctttctcgaacagaggcaggagccaagcgtcgtcaagagaccagcagcagcaaccgtccatccatgagcggccgcaaGAATTCgatatcaagcttatcgataatcaacctctggattacaaaatttgtgaaagattgactggtattcttaactatgttgctccttttacgctatgtggatacgctgctttaatgcctttgtatcatgctattgcttcccgtatggctttcattttctcctccttgtataaatcctggttgctgtctctttatgaggagttgtggcccgttgtcaggcaacgtggcgtggtgtgcactgtgtttgctgacgcaacccccactggttggggcattgccaccacctgtcagctcctttccgggactttcgctttccccctccctattgccacggcggaactcatcgccgcctgccttgcccgctgctggacaggggctcggctgttgggcactgacaattccgtggtgttgtcggggaaATCATcgtcctttccTtggctgctcgcctgtgttgccacctggattctgcgcgggacgtccttctgctacgtcccttcggccctcaatccagcggaccttccttcccgcggcctgctgccggctctgcggcctcttccgcgtcttcgccttcgccctcagacgagtcggatctccctttgggccgcctccccgcatcgataccgtcgacctcgagggggggcccggtaccCGGGAATCAATTCACTCCTCAGGTGCAGGCTGCCTATCAGAAGGTGGTGGCTGGTGTGGCCAATGCCCTGGCTCACAAATACCACTGAGATCTTTTTCCCTCTGCCAAAAATTATGGGGACATCATGAAGCCCCTTGAGCATCTGACTTCTGGCTAATAAAGGAAATTTATTTTCATTGCAATAGTGTGTTGGAATTTTTTGTGTCTCTCACTCGGAAGGACATATGGGAGGGCAAATCATTTAAAACATCAGAATGAGTATTTGGTTTAGAGTTTGGCAACATATGCCCATATGCTGGCTGCCATGAACAAAGGTTGGCTATAAAGAGGTCATCAGTATATGAAACAGCCCCCTGCTGTCCATTCCTTATTCCATAGAAAAGCCTTGACTTGAGGTTAGATTTTTTTTATATTTTGTTTTGTGTTATTTTTTTCTTTAACATCCCTAAAATTTTCCTTACATGTTTTACTAGCCAGATTTTTCCTCCTCTCCTGACTACTCCCAGTCATAGCTGTCCCTCTTCTCTTATGGAGATCCCTCGACCTGCAGCCCAAGCTGTAGATAAGTAGCATGGCGGGTTAATCATTAACTACAAGGAACCCCTAGTGATGGAGTTGGCCACTCCCTCTCTGCGCGCTCGCTCGCTCACTGAGGCCGGGCGACCAAAGGTCGCCCGACGCCCGGGCTTTGCCCGGGCGGCCTCAGTGAGCGAGCGAGCGCGCAGCTGGGCCTCAGTGAGCGAGCGAGCGCGCAGCTGCATTAATGAATCGGCCAACGCGCGGGGAGAGGCGGTTTGCGTATTGGGCGCTCTTCCGCTTCCTCGCTCACTGACTCGCTGCGCTCGGTCGTTCGGCTGCGGCGAGCGGTATCAGCTCACTCAAAGGCGGTAATACGGTTATCCACAGAATCAGGGGATAACGCAGGAAAGAACATGTGAGCAAAAGGCCAGCAAAAGGCCAGGAACCGTAAAAAGGCCGCGTTGCTGGCGTTTTTCCATAGGCTCCGCCCCCCTGACGAGCATCACAAAAATCGACGCTCAAGTCAGAGGTGGCGAAACCCGACAGGACTATAAAGATACCAGGCGTTTCCCCCTGGAAGCTCCCTCGTGCGCTCTCCTGTTCCGACCCTGCCGCTTACCGGATACCTGTCCGCCTTTCTCCCTTCGGGAAGCGTGGCGCTTTCTCATAGCTCACGCTGTAGGTATCTCAGTTCGGTGTAGGTCGTTCGCTCCAAGCTGGGCTGTGTGCACGAACCCCCCGTTCAGCCCGACCGCTGCGCCTTATCCGGTAACTATCGTCTTGAGTCCAACCCGGTAAGACACGACTTATCGCCACTGGCAGCAGCCACTGGTAACAGGATTAGCAGAGCGAGGTATGTAGGCGGTGCTACAGAGTTCTTGAAGTGGTGGCCTAACTACGGCTACACTAGAAGAACAGTATTTGGTATCTGCGCTCTGCTGAAGCCAGTTACCTTCGGAAAAAGAGTTGGTAGCTCTTGATCCGGCAAACAAACCACCGCTGGTAGCGGTGGTTTTTTTGTTTGCAAGCAGCAGATTACGCGCAGAAAAAAAGGATCTCAAGAAGATCCTTTGATCTTTTCTACGGGGTCTGACGCTCAGTGGAACGAAAACTCACGTTAAGGGATTTTGGTCATGAGATTATCAAAAAGGATCTTCACCTAGATCCTTTTAAATTAAAAATGAAGTTTTAAATCAATCTAAAGTATATATGAGTAAACTTGGTCTGACAGTTACCAATGCTTAATCAGTGAGGCACCTATCTCAGCGATCTGTCTATTTCGTTCATCCATAGTTGCCTGACTCCCCGTCGTGTAGATAACTACGATACGGGAGGGCTTACCATCTGGCCCCAGTGCTGCAATGATACCGCGAGACCCACGCTCACCGGCTCCAGATTTATCAGCAATAAACCAGCCAGCCGGAAGGGCCGAGCGCAGAAGTGGTCCTGCAACTTTATCCGCCTCCATCCAGTCTATTAATTGTTGCCGGGAAGCTAGAGTAAGTAGTTCGCCAGTTAATAGTTTGCGCAACGTTGTTGCCATTGCTACAGGCATCGTGGTGTCACGCTCGTCGTTTGGTATGGCTTCATTCAGCTCCGGTTCCCAACGATCAAGGCGAGTTACATGATCCCCCATGTTGTGCAAAAAAGCGGTTAGCTCCTTCGGTCCTCCGATCGTTGTCAGAAGTAAGTTGGCCGCAGTGTTATCACTCATGGTTATGGCAGCACTGCATAATTCTCTTACTGTCATGCCATCCGTAAGATGCTTTTCTGTGACTGGTGAGTACTCAACCAAGTCATTCTGAGAATAGTGTATGCGGCGACCGAGTTGCTCTTGCCCGGCGTCAATACGGGATAATACCGCGCCACATAGCAGAACTTTAAAAGTGCTCATCATTGGAAAACGTTCTTCGGGGCGAAAACTCTCAAGGATCTTACCGCTGTTGAGATCCAGTTCGATGTAACCCACTCGTGCACCCAACTGATCTTCAGCATCTTTTACTTTCACCAGCGTTTCTGGGTGAGCAAAAACAGGAAGGCAAAATGCCGCAAAAAAGGGAATAAGGGCGACACGGAAATGTTGAATACTCATACTCTTCCTTTTTCAATATTATTGAAGCATTTATCAGGGTTATTGTCTCATGAGCGGATACATATTTGAATGTATTTAGAAAAATAAACAAATAGGGGTTCCGCGCACATTTCCCCGAAAAGTGCCACCTGACGTCTAAGAAACCATTATTATCATGACATTAACCTATAAAAATAGGCGTATCACGAGGCCCTTTCGTCTCGCGCGTTTCGGTGATGACGGTGAAAACCTCTGACACATGCAGCTCCCGGAGACGGTCACAGCTTGTCTGTAAGCGGATGCCGGGAGCAGACAAGCCCGTCAGGGCGCGTCAGCGGGTGTTGGCGGGTGTCGGGGCTGGCTTAACTATGCGGCATCAGAGCAGATTGTACTGAGAGTGCACCATATGCGGTGTGAAATACCGCACAGATGCGTAAGGAGAAAATACCGCATCAGGCGATTCCAACATCCAATAAATCATACAGGCAAGGCAAAGAATTAGCAAAATTAAGCAATAAAGCCTCAGAGCATAAAGCTAAATCGGTTGTACCAAAAACATTATGACCCTGTAATACTTTTGCGGGAGAAGCCTTTATTTCAACGCAAGGATAAAAATTTTTAGAACCCTCATATATTTTAAATGCAATGCCTGAGTAATGTGTAGGTAAAGATTCAAACGGGTGAGAAAGGCCGGAGACAGTCAAATCACCATCAATATGATATTCAACCGTTCTAGCTGATAAATTCATGCCGGAGAGGGTAGCTATTTTTGAGAGGTCTCTACAAAGGCTATCAGGTCATTGCCTGAGAGTCTGGAGCAAACAAGAGAATCGATGAACGGTAATCGTAAAACTAGCATGTCAATCATATGTACCCCGGTTGATAATCAGAAAAGCCCCAAAAACAGGAAGATTGTATAAGCAAATATTTAAATTGTAAGCGTTAATATTTTGTTAAAATTCGCGTTAAATTTTTGTTAAATCAGCTCATTTTTTAACCAATAGGCCGAAATCGGCAAAATCCCTTATAAATCAAAAGAATAGACCGAGATAGGGTTGAGTGTTGTTCCAGTTTGGAACAAGAGTCCACTATTAAAGAACGTGGACTCCAACGTCAAAGGGCGAAAAACCGTCTATCAGGGCGATGGCCCACTACGTGAACCATCACCCTAATCAAGTTTTTTGGGGTCGAGGTGCCGTAAAGCACTAAATCGGAACCCTAAAGGGAGCCCCCGATTTAGAGCTTGACGGGGAAAGCCGGCGAACGTGGCGAGAAAGGAAGGGAAGAAAGCGAAAGGAGCGGGCGCTAGGGCGCTGGCAAGTGTAGCGGTCACGCTGCGCGTAACCACCACACCCGCCGCGCTTAATGCGCCGCTACAGGGCGCGTACTATGGTTGCTTTGACGAGCACGTATAACGTGCTTTCCTCGTTAGAATCAGAGCGGGAGCTAAACAGGAGGCCGATTAAAGGGATTTTAGACAGGAACGGTACGCCAGAATCCTGAGAAGTGTTTTTATAATCAGTGAGGCCACCGAGTAAAAGAGTCTGTCCATCACGCAAATTAACCGTTGTCGCAATACTTCTTTGATTAGTAATAACATCACTTGCCTGAGTAGAAGAACTCAAACTATCGGCCTTGCTGGTAATATCCAGAACAATATTACCGCCAGCCATTGCAACGGAATCGCCATTCGCCATTCAGGCTGCGCAACTGTTGGGAAGGGCGATCGGTGCGGGCCTCTTCGCTATTACGCCAGCTGCGCGCTCGCTCGCTCACTGAGGCCG

>pEno2_NSE-mZF-KRAB-mouse-QSGD-WPRE-B11 complete sequence 8298 bp

CCCGGGCAAAGCCCGGGCGTCGGGCGACCTTTGGTCGCCCGGCCTCAGTGAGCGAGCGAGCGCGCAGAGAGGGAGTGGCCAACTCCATCACTAGGGGTTCCTTGTAGTTAATGATTAACCCGCCATGCTACTTATCTACTCGACATTGATTATTGACTagcctcctctgctcgcccaatccttccaaccccctatggtggtatggctgacacagaaaatgtctgctcctgtatgggacatttgcccctcttctccaaatataagacaggatgaggcctagcttttgctgctccaaagttttaaaagaacacattgcacggcatttagggactctaaagggtggaggaggaatgagggaattgcatcatgccaaggctggtcctcatccatcactgcttccagggcccagagtggcttccaggaggtattcttacaaaggaagcccgatctgtagctaacactcagagcccattttcctgcgttaacccctcccgacctcatatacaggagtaacatgatcagtgacctgggggagctggccaaactgcgggacctgcccaagctgagggccttggtgctgctggacaacccctgtgccgatgagactgactaccgccaggaggccctggtgcagatggcacacctagagcgcctagacaaagagtactatgaggacgaggaccgggcagaagctgaggagatccgacagaggctgaaggaggaacaggagcaagaactcgacccggaccaagacatggaaccgtacctcccgccaacttagtggcacctctagcctgcagggacagtaaaggtgatggcaggaaggcagcccccggaggtcaaaggctgggcacgcgggaggagaggccagagtcagaggctgcgggtatctcagatatgaaggaaagatgagagaggctcaggaagaggtaagaaaagacacaagagaccagagaagggagaagaattagagagggaggcagaggaccgctgtctctacagacatagctggtagagactgggaggaagggatgaaccctgagcgcatgaagggaaggaggtggctggtggtatatggaggatgtagctgggccagggaaaagatcctgcactaaaaatctgaagctaaaaataacaggacacggggtggagaggcgaaaggagggcagagtgaggcagagagactgagaggcctggggatgtgggcattccggtagggcacacagttcacttgtcttctctttttccaggaggccaaagatgctgacctcaagaactcataataccccagtggggaccaccgcattcatagccctgttacaagaagtgggagatgttcctttttgtcccagactggaaatccgttacatcccgaggctcaggttctgtggtggtcatctctgtgtggcttgttctgtgggcctacctaaagtcctaagcacagctctcaagcagatccgaggcgactaagatgctagtaggggttgtctggagagaagagccgaggaggtgggctgtgatggatcagttcagctttcaaataaaaaggcgtttttatattctgtgtcgagttcgtgaacccctgtggtgggcttctccatctgtctgggttagtacctgccactatactggaataaggggacgcctgcttccctcgagttggctggacaaggttatgagcatccgtgtacttatggggttgccagcttggtcctggatcgcccgggcccttcccccacccgttcggttccccaccaccacccgcgctcgtacgtgcgtctccgcctgcagctcttgactcatcggggccccccgggtcacatgcgctcgctcggctctataggcgccgccccctgcccacCCCCCGCCCGCGCTGGGAGCCGCAGCCGCCGCCACTCCTGCTCTCTCTGCGCCGCCGCCGTCACCACCGCCACCGCCACCGGCTGAGTCTGCAGTCgctagcctcgagaccATGGGCCGCATTAGAAAGAAACTCAGACTCGCAGAAAGACCTTACGCCTGTCCTGTGGAATCCTGTGATAGACGGTTCAGCCAGAGCGGGGATCTGACAAGGCACATCAGAATTCATACTGGGTCCCAGAAGCCCTTCCAGTGCCGGATCTGTATGCGCAACTTTAGCCAGTCCGGAGACCTCACCCGACACATCCGAACCCATACAGGGGAGAAGCCTTTCGCCTGCGACATTTGTGGTCGGAAATTTGCTCAGAGCGGCGATAGGAAGAGACACACAAAAATCCATACTGGCTCCCAGAAGCCATTCCAGTGCCGAATTTGTATGAGGAATTTTTCTCAGAGTGGCGACCTGACTCGACACATCAGGACTCATACCGGCGAAAAGCCCTTCGCATGCGACATTTGTGGAAGGAAATTTGCCCAGTCTGGGGATCGGAAGCGCCACACCAAAATCCATCTCAGACAGAAGGACGGAGGAGGAGGTTCCGGAGGAGGAGGTAGTGGCGGAGGGGGTTCACAGAAGCCTTTCCAGTGCAGAATCTGTATGCGGAACTTTTCACAGAGCGGAGATCTGACCAGACACATCCGGACACATACTGGGGAGAAGCCATTCGCTTGCGACATTTGTGGTAGGAAATTTGCACAGTCTGGCGATCGAAAGAGGCACACCAAAATCCATACAGGAAGTCAGAAACCTTTCCAGTGCCGCATTTGTATGCGAAATTTTTCCCAGTCTGGTGACCTGACACGCCATATTCGAACCCATACAGGGGAAAAACCTTTCGCCTGTGACATTTGTGGAAGAAAATTTGCTCAGAGCGGGGATAGAAAGCGGCACACTAAAATCCATACCGGCTCTCAGAAACCATTCCAGTGCCGGATTTGTATGCGCAACTTTAGTCAGTCAGGCGACCTGACCAGACACATCAGAACTCACACCGGAGAGAAACCCTTCGCATGTGATATCTGTGGTCGGAAATTCGCCCAGAGCGGCGATCGCAAGCGACACACTAAAATCCACCTCCGCCAGAAGGACGGCGGAggatccggagggggtgggtccgaagagatgctcagttttagagatgtcgctattgacttttcagccgaggaatgggagtgcctggaacctgcccagtggaacctgtacagggacgtgatgctggagaattatagccacctggtcttcctgggcctcgcctcctgcaagccctacctcgtgacctttctcgaacagaggcaggagccaagcgtcgtcaagagaccagcagcagcaaccgtccatccatgagcggccgcaaGAATTCgatatcaagcttatcgataatcaacctctggattacaaaatttgtgaaagattgactggtattcttaactatgttgctccttttacgctatgtggatacgctgctttaatgcctttgtatcatgctattgcttcccgtatggctttcattttctcctccttgtataaatcctggttgctgtctctttatgaggagttgtggcccgttgtcaggcaacgtggcgtggtgtgcactgtgtttgctgacgcaacccccactggttggggcattgccaccacctgtcagctcctttccgggactttcgctttccccctccctattgccacggcggaactcatcgccgcctgccttgcccgctgctggacaggggctcggctgttgggcactgacaattccgtggtgttgtcggggaaATCATcgtcctttccTtggctgctcgcctgtgttgccacctggattctgcgcgggacgtccttctgctacgtcccttcggccctcaatccagcggaccttccttcccgcggcctgctgccggctctgcggcctcttccgcgtcttcgccttcgccctcagacgagtcggatctccctttgggccgcctccccgcatcgataccgtcgacctcgagggggggcccggtaccCGGGAATCAATTCACTCCTCAGGTGCAGGCTGCCTATCAGAAGGTGGTGGCTGGTGTGGCCAATGCCCTGGCTCACAAATACCACTGAGATCTTTTTCCCTCTGCCAAAAATTATGGGGACATCATGAAGCCCCTTGAGCATCTGACTTCTGGCTAATAAAGGAAATTTATTTTCATTGCAATAGTGTGTTGGAATTTTTTGTGTCTCTCACTCGGAAGGACATATGGGAGGGCAAATCATTTAAAACATCAGAATGAGTATTTGGTTTAGAGTTTGGCAACATATGCCCATATGCTGGCTGCCATGAACAAAGGTTGGCTATAAAGAGGTCATCAGTATATGAAACAGCCCCCTGCTGTCCATTCCTTATTCCATAGAAAAGCCTTGACTTGAGGTTAGATTTTTTTTATATTTTGTTTTGTGTTATTTTTTTCTTTAACATCCCTAAAATTTTCCTTACATGTTTTACTAGCCAGATTTTTCCTCCTCTCCTGACTACTCCCAGTCATAGCTGTCCCTCTTCTCTTATGGAGATCCCTCGACCTGCAGCCCAAGCTGTAGATAAGTAGCATGGCGGGTTAATCATTAACTACAAGGAACCCCTAGTGATGGAGTTGGCCACTCCCTCTCTGCGCGCTCGCTCGCTCACTGAGGCCGGGCGACCAAAGGTCGCCCGACGCCCGGGCTTTGCCCGGGCGGCCTCAGTGAGCGAGCGAGCGCGCAGCTGGGCCTCAGTGAGCGAGCGAGCGCGCAGCTGCATTAATGAATCGGCCAACGCGCGGGGAGAGGCGGTTTGCGTATTGGGCGCTCTTCCGCTTCCTCGCTCACTGACTCGCTGCGCTCGGTCGTTCGGCTGCGGCGAGCGGTATCAGCTCACTCAAAGGCGGTAATACGGTTATCCACAGAATCAGGGGATAACGCAGGAAAGAACATGTGAGCAAAAGGCCAGCAAAAGGCCAGGAACCGTAAAAAGGCCGCGTTGCTGGCGTTTTTCCATAGGCTCCGCCCCCCTGACGAGCATCACAAAAATCGACGCTCAAGTCAGAGGTGGCGAAACCCGACAGGACTATAAAGATACCAGGCGTTTCCCCCTGGAAGCTCCCTCGTGCGCTCTCCTGTTCCGACCCTGCCGCTTACCGGATACCTGTCCGCCTTTCTCCCTTCGGGAAGCGTGGCGCTTTCTCATAGCTCACGCTGTAGGTATCTCAGTTCGGTGTAGGTCGTTCGCTCCAAGCTGGGCTGTGTGCACGAACCCCCCGTTCAGCCCGACCGCTGCGCCTTATCCGGTAACTATCGTCTTGAGTCCAACCCGGTAAGACACGACTTATCGCCACTGGCAGCAGCCACTGGTAACAGGATTAGCAGAGCGAGGTATGTAGGCGGTGCTACAGAGTTCTTGAAGTGGTGGCCTAACTACGGCTACACTAGAAGAACAGTATTTGGTATCTGCGCTCTGCTGAAGCCAGTTACCTTCGGAAAAAGAGTTGGTAGCTCTTGATCCGGCAAACAAACCACCGCTGGTAGCGGTGGTTTTTTTGTTTGCAAGCAGCAGATTACGCGCAGAAAAAAAGGATCTCAAGAAGATCCTTTGATCTTTTCTACGGGGTCTGACGCTCAGTGGAACGAAAACTCACGTTAAGGGATTTTGGTCATGAGATTATCAAAAAGGATCTTCACCTAGATCCTTTTAAATTAAAAATGAAGTTTTAAATCAATCTAAAGTATATATGAGTAAACTTGGTCTGACAGTTACCAATGCTTAATCAGTGAGGCACCTATCTCAGCGATCTGTCTATTTCGTTCATCCATAGTTGCCTGACTCCCCGTCGTGTAGATAACTACGATACGGGAGGGCTTACCATCTGGCCCCAGTGCTGCAATGATACCGCGAGACCCACGCTCACCGGCTCCAGATTTATCAGCAATAAACCAGCCAGCCGGAAGGGCCGAGCGCAGAAGTGGTCCTGCAACTTTATCCGCCTCCATCCAGTCTATTAATTGTTGCCGGGAAGCTAGAGTAAGTAGTTCGCCAGTTAATAGTTTGCGCAACGTTGTTGCCATTGCTACAGGCATCGTGGTGTCACGCTCGTCGTTTGGTATGGCTTCATTCAGCTCCGGTTCCCAACGATCAAGGCGAGTTACATGATCCCCCATGTTGTGCAAAAAAGCGGTTAGCTCCTTCGGTCCTCCGATCGTTGTCAGAAGTAAGTTGGCCGCAGTGTTATCACTCATGGTTATGGCAGCACTGCATAATTCTCTTACTGTCATGCCATCCGTAAGATGCTTTTCTGTGACTGGTGAGTACTCAACCAAGTCATTCTGAGAATAGTGTATGCGGCGACCGAGTTGCTCTTGCCCGGCGTCAATACGGGATAATACCGCGCCACATAGCAGAACTTTAAAAGTGCTCATCATTGGAAAACGTTCTTCGGGGCGAAAACTCTCAAGGATCTTACCGCTGTTGAGATCCAGTTCGATGTAACCCACTCGTGCACCCAACTGATCTTCAGCATCTTTTACTTTCACCAGCGTTTCTGGGTGAGCAAAAACAGGAAGGCAAAATGCCGCAAAAAAGGGAATAAGGGCGACACGGAAATGTTGAATACTCATACTCTTCCTTTTTCAATATTATTGAAGCATTTATCAGGGTTATTGTCTCATGAGCGGATACATATTTGAATGTATTTAGAAAAATAAACAAATAGGGGTTCCGCGCACATTTCCCCGAAAAGTGCCACCTGACGTCTAAGAAACCATTATTATCATGACATTAACCTATAAAAATAGGCGTATCACGAGGCCCTTTCGTCTCGCGCGTTTCGGTGATGACGGTGAAAACCTCTGACACATGCAGCTCCCGGAGACGGTCACAGCTTGTCTGTAAGCGGATGCCGGGAGCAGACAAGCCCGTCAGGGCGCGTCAGCGGGTGTTGGCGGGTGTCGGGGCTGGCTTAACTATGCGGCATCAGAGCAGATTGTACTGAGAGTGCACCATATGCGGTGTGAAATACCGCACAGATGCGTAAGGAGAAAATACCGCATCAGGCGATTCCAACATCCAATAAATCATACAGGCAAGGCAAAGAATTAGCAAAATTAAGCAATAAAGCCTCAGAGCATAAAGCTAAATCGGTTGTACCAAAAACATTATGACCCTGTAATACTTTTGCGGGAGAAGCCTTTATTTCAACGCAAGGATAAAAATTTTTAGAACCCTCATATATTTTAAATGCAATGCCTGAGTAATGTGTAGGTAAAGATTCAAACGGGTGAGAAAGGCCGGAGACAGTCAAATCACCATCAATATGATATTCAACCGTTCTAGCTGATAAATTCATGCCGGAGAGGGTAGCTATTTTTGAGAGGTCTCTACAAAGGCTATCAGGTCATTGCCTGAGAGTCTGGAGCAAACAAGAGAATCGATGAACGGTAATCGTAAAACTAGCATGTCAATCATATGTACCCCGGTTGATAATCAGAAAAGCCCCAAAAACAGGAAGATTGTATAAGCAAATATTTAAATTGTAAGCGTTAATATTTTGTTAAAATTCGCGTTAAATTTTTGTTAAATCAGCTCATTTTTTAACCAATAGGCCGAAATCGGCAAAATCCCTTATAAATCAAAAGAATAGACCGAGATAGGGTTGAGTGTTGTTCCAGTTTGGAACAAGAGTCCACTATTAAAGAACGTGGACTCCAACGTCAAAGGGCGAAAAACCGTCTATCAGGGCGATGGCCCACTACGTGAACCATCACCCTAATCAAGTTTTTTGGGGTCGAGGTGCCGTAAAGCACTAAATCGGAACCCTAAAGGGAGCCCCCGATTTAGAGCTTGACGGGGAAAGCCGGCGAACGTGGCGAGAAAGGAAGGGAAGAAAGCGAAAGGAGCGGGCGCTAGGGCGCTGGCAAGTGTAGCGGTCACGCTGCGCGTAACCACCACACCCGCCGCGCTTAATGCGCCGCTACAGGGCGCGTACTATGGTTGCTTTGACGAGCACGTATAACGTGCTTTCCTCGTTAGAATCAGAGCGGGAGCTAAACAGGAGGCCGATTAAAGGGATTTTAGACAGGAACGGTACGCCAGAATCCTGAGAAGTGTTTTTATAATCAGTGAGGCCACCGAGTAAAAGAGTCTGTCCATCACGCAAATTAACCGTTGTCGCAATACTTCTTTGATTAGTAATAACATCACTTGCCTGAGTAGAAGAACTCAAACTATCGGCCTTGCTGGTAATATCCAGAACAATATTACCGCCAGCCATTGCAACGGAATCGCCATTCGCCATTCAGGCTGCGCAACTGTTGGGAAGGGCGATCGGTGCGGGCCTCTTCGCTATTACGCCAGCTGCGCGCTCGCTCGCTCACTGAGGCCG
