# Supplementary material for: Deimmunization for gene therapy: host matching of synthetic zinc finger constructs enables long-term mutant Huntingtin repression in mice
Source: Mol Neurodegener. 2016 Sep 6;11(1):64. doi: 10.1186/s13024-016-0128-x (PMC5013590; doi:10.1186/s13024-016-0128-x)
Supplement: Additional file 2: — Summary of mice injected with ZF-KOX1, mZF-KRAB, GFP or PBS. (DOCX 48 kb) [file 13024_2016_128_MOESM2_ESM.docx]

**Additional file 2. Summary of number of mice injected with ZF-KOX-1, mZF-KRAB, GFP or PBS.**

| **Experiment** | **Treatment** | **Genotype** | **Weeks post-injection** | **n** |
| --- | --- | --- | --- | --- |
| Histology analysis: inflammatory responses and neuronal loss | ZF-KOX1 | WT | 4 | 4 |
|  |  |  | 6 | 4 |
|  | mZF-KRAB |  | 4 | 4 |
|  |  |  | 6 | 4 |
|  | GFP |  | 4 | 4 |
|  |  |  | 6 | 4 |
|  | PBS |  | 4 | 3 |
|  |  |  | 6 | 3 |
| Gene expression analysis | ZF-KOX1 | R6/2 | 2 | 3 |
|  |  |  | 4 | 3 |
|  |  |  | 6 | 3 |
|  | mZF-KRAB | R6/1 | 2 | 6 |
|  |  |  | 4 | 7 |
|  |  |  | 6 | 7 |
