# Supplementary material for: Deimmunization for gene therapy: host matching of synthetic zinc finger constructs enables long-term mutant Huntingtin repression in mice
Source: Mol Neurodegener. 2016 Sep 6;11(1):64. doi: 10.1186/s13024-016-0128-x (PMC5013590; doi:10.1186/s13024-016-0128-x)
Supplement: Additional file 3: — Primers used in qRT-PCR analyses. (DOCX 100 kb) [file 13024_2016_128_MOESM3_ESM.docx]

**Additional file 3. Primers used in qRT-PCR analyses.**

CAG-repeat number per gene and corresponding primer sets for qRT-PCR. Name prefixes: mut=mutant; m=mouse. Approximate CAG repeat number for wild-type genes was obtained from Genbank mRNA data. CAG-repeat length: the first number corresponds to pure CAG repeats, the second number to broken CAG repeats (containing CAA or CAT).

For Fig 7:

| **Gene** | **CAG repeat length** | **Forward primer** | **Reverse primer** |
| --- | --- | --- | --- |
| ZF-KOX1 | N/A | GTGGAAGCTGCTGGACACT | AACGTAAAGTGACCGGGGCCG |
| mZF-KRAB | N/A | GGTCCGAAGAGATGCTCAGT | CAGGAAGACCAGGTGGCTAT |
|  |  |  |  |
| mut HTT | ~150 | GCTGCACCGACCGTGAGT | CGCAGGCTGCAGGGTTAC |
|  |  |  |  |
| mHtt | 4,7 | CAGATGTCAGAATGGTGGCT | GCCTTGGAAGATTAGAATCCA |
| mATN1 | 3,10 | CACCTGCCTCCACCTCATGGC | ATGCTCCTTGGGGGCCCTGG |
| mATXN2 | 6,10 | ATCCCAATGCAAAGGAGTTC | CTGCTGATGACCCACCATAG |
| mTBP | 3,13 | ACTTCGTGCAAGAAATGCTG | GCTCATAGCTCTTGGCTCCT |
| mHPRT | N/A | GGTTAAGCAGTACAGCCCCA | AGAGGTCCTTTTCACCAGCA |
| mActb | N/A | GCTTCTTTGCAGCTCCTTCGT | CCAGCGCAGCGATATCG |
| mAtp5b | N/A | CCACCGACATGGGCACAATGCA | ATGGGCAAAGGTGGTTGCAGGG |

For Fig 8:

| **Primer’s name** | **Sequence 5’-3’** | **Application** | **Reference** |
| --- | --- | --- | --- |
| R6/1 Forward | CGCAGGCTAGGGCTGTCAATCATGCT | Genotyping | Mielcarek et al PLoS Biology 2013 |
| R6/1 Reverse | TCATCAGCTTTTCCAGGGTCGCCAT | Genotyping | Mielcarek et al PLoS Biology 2013 |
| Mutant HTT Forward | GCTGCACCGACCGTGAGT | Taq-man qPCR | Mielcarek et al PLoS Biology 2013 |
| Mutant HTT Reverse | CGCAGGCTGCAGGGTTAC | Taq-man qPCR | Mielcarek et al PLoS Biology 2013 |
| Mutant HTT Probe | [6FAM]CAGCTCCCTGTCCCGGCGG[TAM] | Taq-man qPCR | Mielcarek et al PLoS Biology 2013 |
| HTT Forward | CTCAGAAGTGCAGGCCTTACCT | Taq-man qPCR | Mielcarek et al PLoS Biology 2013 |
| HTT Reverse | GATTCCTCCGGTCTTTTGCTT | Taq-man qPCR | Mielcarek et al PLoS Biology 2013 |
| HTT-Probe | [6FAM]CAGCTCCCTGTCCCGGCGG[TAM] | Taq-man qPCR | Mielcarek et al PLoS Biology 2013 |
| ZF Forward | TACCTTACAGCGCCATACCA | Taq-man qPCR | New |
| ZF Reverse | GGCTAAAGTTGCGCATACAA | Taq-man qPCR | New |
| ZF Probe | [6FAM]CTAGTTGTGAGCCGCCGCCA[TAM] | Taq-man qPCR | New |
