# Supplementary material for: Deimmunization for gene therapy: host matching of synthetic zinc finger constructs enables long-term mutant Huntingtin repression in mice
Source: Mol Neurodegener. 2016 Sep 6;11(1):64. doi: 10.1186/s13024-016-0128-x (PMC5013590; doi:10.1186/s13024-016-0128-x)
Supplement: Additional file 4: — Identification of reference genes for qPCR from whole brain of the R6/1 and WT mice. (DOCX 51 kb) [file 13024_2016_128_MOESM4_ESM.docx]

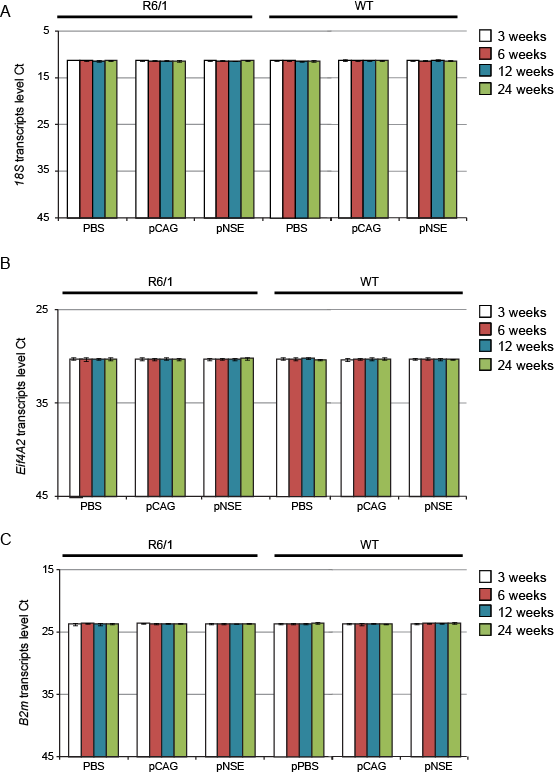


**Additional file 5. Identification of reference genes for qPCR from whole brain of the R6/1 and WT mice.** To find reference genes spanning all exercised time points (3, 6, 12 and 24 weeks) GeNorm analysis was used. Raw crossing threshold (Ct) data for a panel of positively-identified genes from the geNorm kit in WT and R6/1 mice are presented from **(A)** *18S* (18S rRNA, 19791) **(B)** *Eif4A2* (Eukaryotic translation initiation factor 4A2, 13682) **(C)** *B2m*, (Beta-2-microglobulin, 12010). Error bars are ± SEM (n = 3).
